# Clinical impact of endemic NDM-producing *Klebsiella pneumoniae* in intensive care units of the national referral hospital in Jakarta, Indonesia

**DOI:** 10.1186/s13756-020-00716-7

**Published:** 2020-05-11

**Authors:** Yulia Rosa Saharman, Anis Karuniawati, Rudyanto Sedono, Dita Aditianingsih, Wil H. F. Goessens, Corné H. W. Klaassen, Henri A. Verbrugh, Juliëtte A. Severin

**Affiliations:** 1 Department of Clinical Microbiology, Faculty of Medicine, Universitas Indonesia / Dr. Cipto Mangunkusumo General Hospital, Jakarta, Indonesia; 2 Department of Medical Microbiology and Infectious Diseases, Erasmus MC University Medical Center Rotterdam, Dr. Molewaterplein 40, 3015 GD Rotterdam, The Netherlands; 3 Critical Care Division, Department of Anesthesia and Intensive Care, Faculty of Medicine, Universitas Indonesia / Dr. Cipto Mangunkusumo General Hospital, Jakarta, Indonesia

**Keywords:** *Klebsiella pneumoniae*, Microbial drug resistance, Carbapenemase, Intensive care unit, Mortality, Indonesia

## Abstract

**Objective:**

A prospective observational study was performed to assess the epidemiology and clinical impact of carbapenem-non-susceptible *Klebsiella pneumoniae* (CNKP) in intensive care units (ICUs) of the national referral hospital in Jakarta, Indonesia.

**Materials/methods:**

Adult patients consecutively hospitalized for > 48 h in two ICUs of the national referral hospital were included from April until October 2013 and from April until August 2014. *K. pneumoniae* from clinical cultures and standardized screening of rectum and throat on admission, discharge and weekly if hospitalized > 7 days were collected. Environmental niches and healthcare workers (HCWs) were also screened. Susceptibility was determined phenotypically and the presence of carbapenemase genes by PCR. Raman spectroscopy as well as multiple-locus variable number tandem repeat analysis (MLVA) were used for typing.

**Results:**

Twenty-two out of 412 (5.3%) patients carried CNKP on admission and 37/390 (9.5%) acquired CNKP during ICU stay. The acquisition rate was 24.7/1000 patient-days at risk. One out of 31 (3.2%) environmental isolates was a CNKP. None of the HCWs carried CNKP. Acquisition of CNKP was associated with longer ICU stay (adjusted Hazard Ratio: 2.32 [CI_99_: 1.35–3.68]). ICU survival was lower among patients with CNKP compared to patients with carbapenem-susceptible *K. pneumoniae* (aHR 2.57, *p* = 0.005). Ninety-six of the 100 (96%) CNKP isolates carried a carbapenemase gene, predominantly *bla*_NDM_. Raman typing revealed three major clusters among 48 Raman types identified, whereas MLVA distinguished six major clusters among a total of 30 different genotypes.

**Conclusions:**

NDM-producing CNKP are introduced into these ICUs and some strains expand clonally among patients and the environment, resulting in endemic CNKP. CNKP acquisition was associated with prolonged ICU stay and may affect ICU survival.

**Trial registration:**

The study was registered at Netherlands Trial Register http://www.trialregister.nl. Candidate number: 23527, NTR number: NTR5541, NL number: NL5425 (https://www.trialregister.nl/trial/5424), Retrospectively registered: NTR: 22 December 2015.

## Introduction

Carbapenems are the antibiotics of choice for treatment of life-threatening infections due to multidrug-resistant Gram-negative bacilli. However, the worldwide emergence of carbapenem-non-susceptible *Klebsiella pneumoniae*, especially in intensive care units (ICUs), has become a major challenge. Non-susceptibility to carbapenems in *K. pneumoniae* may be due to production of Ambler class A β-lactamases (e.g. KPC), class B metallo-β-lactamases (MBLs, e.g. VIM, IMP, NDM) or class D oxacillinases (e.g. OXA-48 like enzymes) [1–3].

Although carbapenemase-producing *K. pneumoniae* have emerged globally, geographic variations do exist. *K. pneumoniae* producing KPCs have initially mainly been reported in the USA and Israel, but more recently also from China and Taiwan [1, 2, 4, 5]. *K. pneumoniae* strains carrying OXA-48-like carbapenemases were first described in Turkey in 2003. Currently, *K. pneumoniae* with OXA-48-like carbapenemases are spreading rapidly in many European countries, in addition to being endemic in the Middle East and in Northern Africa [1, 4, 5]. Bacteria with the New Delhi metallo-β-lactamase (NDM) enzyme, which was first identified in Sweden from a patient who had travelled from New Delhi, India, have attained endemic levels in countries of the Indian subcontinent including India, Pakistan, Bangladesh and Sri Lanka [1, 4–8]. This gene is also encountered in bacteria, including *K. pneumoniae*, in some countries in the South East Asian region, including Singapore [9], Thailand [10] and Vietnam [1]. However, so far there have been few data on the epidemiology of carbapenem-non-susceptible *K. pneumoniae* reported from Indonesia, the fourth most populous country in the world. In 2011, 27.6% of the Enterobacteriaceae isolated from specimens at two ICUs in Jakarta was carbapenem-resistant, including one *K. pneumoniae* harboring the *bla*_NDM_ gene [11]. In 2014–2015, the prevalence of resistance to meropenem among *K. pneumoniae* from urinary tract infections in clinical and outpatient clinical settings was 14.0%, but no further analysis of these isolates was performed [12]. The aim of the present study was to delineate the clinical and molecular epidemiology of carbapenem-non-susceptible *K. pneumoniae* isolated in two ICUs of the Dr. Cipto Mangunkusumo General Hospital, the national referral teaching hospital in Jakarta.

## Materials and methods

### Study design

A prospective observational study was performed in a 1000-bed national referral teaching hospital with 34,000 admissions per year in Jakarta, Indonesia, from April until October 2013 and from April until August 2014. Two ICUs participated, the adult ICU and the Emergency Room (ER)-ICU, with 865 and 390 admissions in 2013, respectively, and 1154 and 439 admissions in 2014, respectively. The adult ICU is a 12-bed open ward with mechanical ventilation facilities, admitting patients with various medical and surgical indications, and one designated nurse per patient during morning shifts and a 1:1.5 nurse/patient ratio during other shifts. It is also used as post-anaesthetic care unit. The ER-ICU has the same design, but 8 beds, and the nurse per patient ratio in the morning shifts is 1:1 and during the other shifts 1:2. This ICU is also used for short observations.

All adult patients (≥18 years old) admitted to one of the two ICUs and hospitalized for more than 48 h were eligible for enrollment in this study. Informed consent was obtained from the patient or their relatives as applicable. Demographic and clinical characteristics such as age, gender, medical or surgical indication, underlying diseases, hospitalization history, and previous use of antibiotics on admission were recorded.

Systemic inflammatory response syndrome (SIRS) criteria on admission were used as a screening tool to assess (severity of) septic illness. The “Acute Physiology and Chronic Health Evaluation II” score was not feasible in this low-resource setting. SIRS is defined as two or more of the following: fever > 38 °C or < 36 °C, heart rate > 90 beats per minute, respiratory rate > 20 breaths per minute or PaCO2 < 32 mmHg, abnormal white blood cell count (> 12,000/mm^3^ or < 4000/mm^3^ or > 10% bands) [13].

The quick Sequential Organ Failure Assessment (qSOFA) score is a newer bedside prompt that may identify patients with suspected infection and helps to determine sepsis in all healthcare environments. The qSOFA score assigns one point for each of the following conditions: systolic blood pressure ≤ 100 mmHg, respiratory rate ≥ 22 breaths per minute, and altered mentation (Glasgow coma scale < 15). The score ranges from 0 to 3 points. A qSOFA score ≥ 2 at the onset of infection is associated with a greater risk of death and prolonged ICU stay. This score was included as well [13].

Acquisition is defined as a screening culture (throat or rectum/stool) or clinical culture with a first detection of *K. pneumoniae* with reduced susceptibility to a carbapenem, that was not present in cultures taken from the patient on admission or in the first 48 h of admission. Outcome measures were acquisition of a carbapenem-susceptible and carbapenem-non-susceptible *K. pneumoniae* (independent of resistance to other classes of antibiotics), length of stay in the ICU, and mortality during ICU stay.

Environmental samples were taken twice (in October 2013 and December 2014), simultaneously in both ICUs. Screening of healthcare workers (HCWs) was performed once. HCWs were defined as all personnel including doctors, nurses and other people (cleaning staff, administration staff, porters, nutritionist) working in one of the two ICUs during the study period.

### Sampling

From patients enrolled, screening cultures were obtained from throat and rectum or stools by experienced ICU nurses on the day of admission, at the time of discharge from the ICU, and weekly if the patient was admitted for seven days or more. The samples were collected with sterile cotton-tipped swabs and placed in Amies transport medium (Oxoid, Basingstoke, UK). The swabs were transported in clean, closed boxes at ambient temperature to the laboratory on the same day. All swabs were processed in the laboratory within 24 h.

Clinical samples were collected from a patient when the ICU physician suspected the patient of having an infection. Specimens were taken under aseptic precautions from the lower respiratory tract, blood, urine, tissue, or wound, on indication.

Environmental samples were taken from various sites, including wash basins, bed rails, bedside cabinet tables, ventilators, and monitor screens (Supplementary Table 1), with sterile cotton-tipped swabs and placed in Amies transport medium [14].

All HCWs working in one of the ICUs were sampled (rectal and throat) once over the course of one month (September 2013) with sterile cotton-tipped swabs, which were transported to the laboratory in Amies transport medium.

### Microbiological methods

#### Isolation and identification of bacteria

In the laboratory, each screening swab was placed in a trypticase soy broth (TSB) supplemented with cefotaxime 2 mg/L plus vancomycin 50 mg/L and incubated overnight. The next day, a loop of broth was sub-cultured on MacConkey agar (Oxoid).

Blood cultures were collected in BACTEC (BD, Franklin Lakes, NJ, USA) bottles as per manufacturer’s instructions with a minimum of 10 mL of blood collected from at least two puncture sites. Other clinical specimens were inoculated onto blood and MacConkey agar plates (Oxoid) and incubated for 24 h at 37 °C. All morphologically different colonies were examined by Gram stain and identified using the VITEK2® system (bioMérieux, Lyon, France).

Strains were stored in duplicate in − 80 °C in TSB with glycerol 10%. One tube of each strain was sent to the Department of Medical Microbiology and Infectious Diseases, Erasmus MC, Rotterdam, the Netherlands, for further analysis. The other tube of each strain remained in the Indonesian laboratory. In the Netherlands, the identity of strains was confirmed using matrix-assisted laser desorption/ionisation (Maldi Biotyper, Bruker Microflex LT, Bruker, London, UK).

The quality control strains used for this part of the study in Indonesia were *Escherichia coli* ATCC 25922 and *Pseudomonas aeruginosa* ATCC 27853, in the laboratory of Erasmus MC multiple quality control strains were used.

#### Antimicrobial susceptibility testing

Imipenem and meropenem susceptibility tests on isolates from screening cultures were performed by standard Kirby-Bauer disc diffusion technique using Mueller-Hinton agar plates (BD) based on EUCAST Disc Diffusion Method for Antimicrobial Susceptibility Testing- Version 3.0 (April 2013). Minimum inhibitory concentrations (MICs) of antibiotics were determined by VITEK2® for clinical isolates. Carbapenem MICs and zone sizes were interpreted according to EUCAST (2013) using the following breakpoints for non-susceptibility: meropenem > 0.25 mg/L (< 24 mm), imipenem > 1 mg/L (< 22 mm) [15].

For this part of the study, quality control strains as described above were used.

#### String test

In order to determine hyper-muco-viscosity, the string assay was performed for all *K. pneumoniae* isolates. For this, the strains were inoculated onto 5% sheep blood agar (BD) and kept overnight at 37 °C_._ An individual colony was then touched with a 1 μL disposable loop which was subsequently pulled up slowly. The string test was deemed positive if a string of ≥5 mm was formed between the colony and the loop [16].

#### Phenotypic detection of carbapenemase

A phenotypic detection test for Ambler class A and B and OXA-48-like carbapenemases was performed with discs (Rosco Diagnostica A/S, Taastrup, Denmark) containing meropenem (10 μg), temocillin (30 μg), meropenem + phenyl boronic acid (PBA), meropenem + dipicolinic acid (DPA), meropenem + PBA + DPA, and meropenem + cloxacillin (CL), using a 0.5 McFarland suspension of the isolates on Mueller Hinton II agar plates. Zone diameters were measured after overnight incubation at 37 °C. The temocillin zone diameter was only interpreted if no synergy was observed with DPA and/or PBA. Isolates without synergy with the PBA or DPA test and a temocillin zone diameter ≤ 10 mm (i.e. the absence of an inhibition zone around the temocillin disc) were considered OXA carbapenemase positive. The interpretation of the PBA and DPA synergy tests and the temocillin disc diffusion were as described previously [17].

#### DNA extraction and PCR for carbapenemase genes

DNA from the isolates was extracted by a cell lysis step and boiling using the InstaGene Matrix (Bio-Rad Laboratories, USA) according to the manufacturer’s instructions. PCR-based detection of Ambler class A carbapenemases (*bla*_KPC_), Ambler class B metallo-β-lactamases (*bla*_NDM_), and class D β-lactamases (*bla*_OXA-48-like_) were carried out using T3000 Thermocycler (Biometra-Whatman, Goettingen). PCR primers and reaction conditions for PCR were as described previously [18–20]. Amplified PCR products were resolved by electrophoresis at 250 V for 30 min on 1.5% agarose gels with 0.5 x Tris (89 mM)-boric acid (89 mM)-EDTA (2 mM) buffer containing SyBr® Safe DNA Gel Stain and visualized under UV light and photographed. In each run, a positive and negative control was included.

#### Clonal relatedness

Raman spectroscopy (SpectraCell RA® Bacterial Strain Analyzer, RiverD International BV, Rotterdam, The Netherlands) was applied as a first typing method [21, 22]. All isolates were grown overnight on trypticase soy agar (TSA; BD). Samples were prepared and submitted to spectrometry as described previously [22]. Raman light scatterings were analyzed by SpectraCell*RA* software version 1.9.0.13444:24 (RiverD). The similarity between pairs of spectra was calculated using the squared Pearson correlation coefficient (R^2^-values), multiplied by 100 and expressed as a percentage. The similarity threshold for this study was set at 91% so that two isolates with an R^2^ below this threshold were considered to be different and were designated different Raman types. Two isolates with an R^2^-value above 99.5% were considered indistinguishable and were considered to have the same Raman type. In case of an R^2^-value between of 91 and 99.5%, these isolates were considered highly related but not identical [23].

Correlation matrices displayed as 2D plots diagram were created using MATLAB version 7.1 (The MathWorks, Natick, MA, USA).

Multiple-locus variable number tandem repeat analysis (MLVA) was used as a second typing method. The MLVA typing protocol was based on Brink et al. [24] with minor modifications (for details, see Supplement). DNA was quantitated using PicoGreen dsDNA reagent (Invitrogen, Bleiswijk, The Netherlands). Amplification reactions contained approximately 1 ng of DNA and primers according to Supplementary Table 2 in 1x Roche FastStart PCR Master Mix (Roche diagnostics, Almere, The Netherlands). The thermocycling protocol consisted of an initial denaturation for 5 min at 95 °C followed by 30 amplification cycles of denaturation for 30s at 95 °C, 30s annealing at 58 °C and 1 min extension at 72 °C. A final extension step of 30 min at 72 °C was applied before reactions were cooled to room temperature. Before loading, amplification products were diluted 100x, combined with the GeneScan 600 LIZ Dye Size Standard (ThermoFisher Scientific, Bleiswijk, The Netherlands) and run on an ABI 3130 capillary electrophoresis platform (ThermoFisher Scientific) using recommended conditions. Electropherograms were analyzed using the MLVA plugin in BioNumerics v7.6 software (Applied-Maths, Sint-Martens-Latem, Belgium). Assignment of repeat numbers was calibrated by comparing our results to those obtained with selected isolates that were genotyped by the Maastricht lab. Typing data was analyzed categorically.

### Statistical analysis

Statistical analyses were performed using SPSS Version 24.0 (SPSS, Chicago, IL, USA). Baseline characteristics from patients admitted to the adult ICU were compared to those in the ER-ICU using Chi square and Mann-Whitney as appropriate. One-way ANOVA was used to compare patient characteristics according to their *K. pneumoniae* status. Univariate and multivariate analyses were performed to establish risk factors associated with in-ICU mortality using a multivariate logistic regression model with backward selection and inclusion of variables with a *p* value < 0.1 in the univariate analysis. Cox proportional regression was used to analyze risk factors for length of stay. Kaplan-Meier method was performed to construct survival curves. The R-code (R-3.6.2.pkg. binary for OS X 10.11 software can be obtained via CRAN, the Comprehensive R Archive Network, http://cran.R-project.org) was used to calculate the competing risks estimates (competing risk analysis is available in an add-on package called cmprsk) of the cumulative incidence function and conditional probability function for ICU discharge and in-ICU mortality [25, 26]. *P* values less than 0.01 were considered significant [27].

## Results

### Patient characteristics and outcomes

During the 11-month study period, 1211 patients were hospitalized in the ICUs (Adult ICU: 863, ER-ICU: 348). Of the 412 included patients, 188 were admitted to the adult ICU and 224 to the ER-ICU. Supplementary Table 3 shows baseline characteristics of included patients in each ICU. There were no significant differences in characteristics between patients in both ICUs, except that in the adult ICU most of the patients had been referred from another ward in the same hospital and the proportion of patients with malignancies was higher. Therefore, we analyzed the data from the ICUs both separately and pooled.

Overall, 192/412 (46.6%) patients had at least one positive culture with *K. pneumoniae,* the remaining 220 patients were free from *K. pneumoniae* on admission and remained so during their ICU stay. One hundred (24.3%) patients already carried *K. pneumoniae* on the day of admission, of whom 78 carried a carbapenem–susceptible *K. pneumoniae* and 22 (5.3%) carried a carbapenem-non-susceptible *K. pneumoniae* strain (Supplementary Figure 1). One hundred patients (32.1%) acquired *K. pneumoniae* during ICU stay, a carbapenem-non-susceptible *K. pneumoniae* strain in 37 cases and a carbapenem-susceptible strain of *K. pneumoniae* by 63 patients. Thus, a total of 59 patients (14.3%) carried a carbapenem-non-susceptible *K. pneumoniae* at a certain moment during their ICU stay. In 44 patients, this *K. pneumoniae* was only found in a screening culture, in five patients only from a clinical specimen, and in ten patients from both screening and clinical samples.

**Fig. 1:**
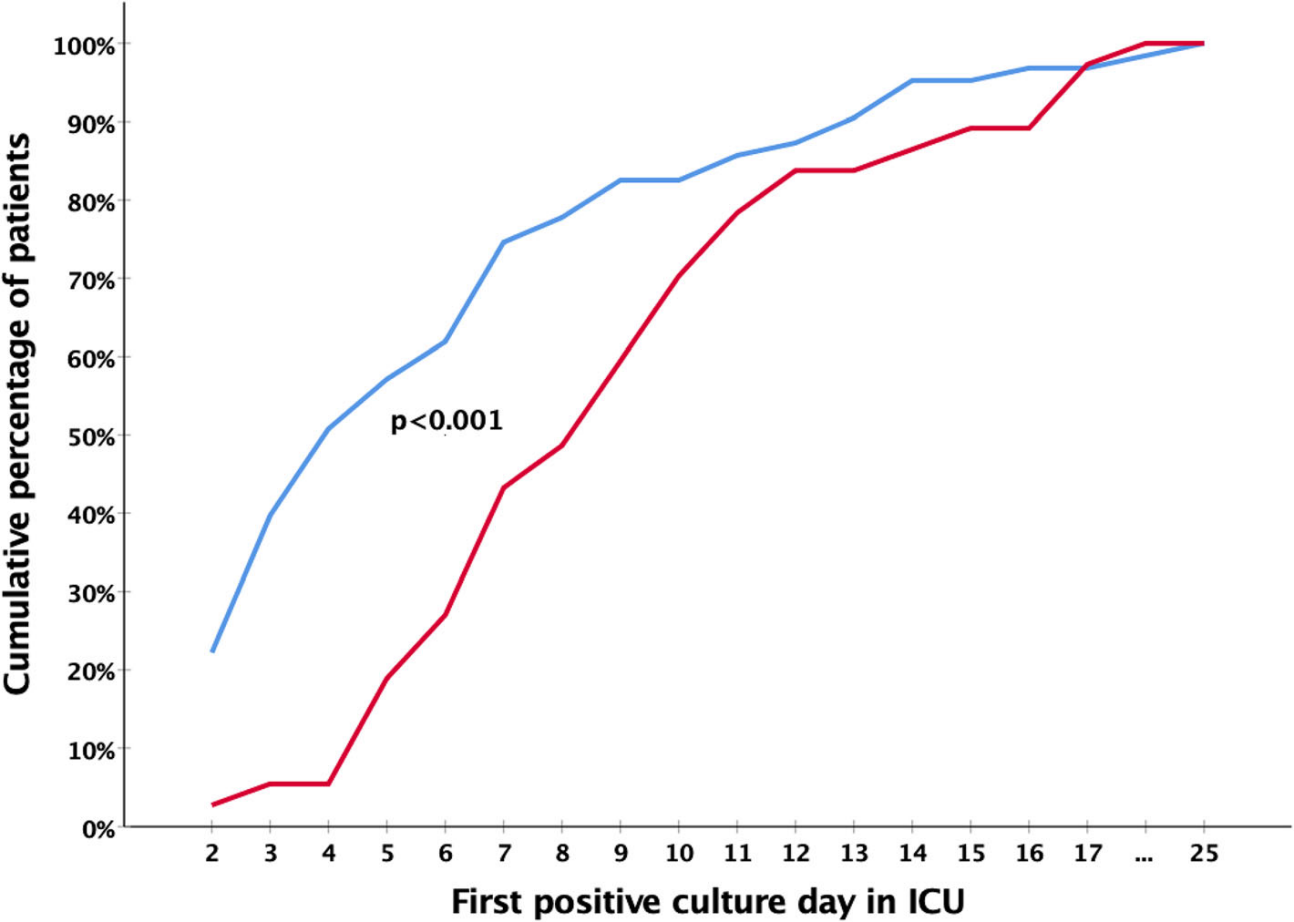
Rate of acquisition of carbapenem-susceptible and -non-susceptible *K. pneumoniae* in ICUs. Acquisition dynamics of carbapenem-susceptible and –non-susceptible *K. pneumoniae* during ICU stay. The blue line represents the cumulative percentage of patients by first day of culture being positive for carbapenem-susceptible *K. pneumoniae* during ICU stay. The red line represents the cumulative percentage of patients by first day of culture being positive for carbapenem-non-susceptible *K. pneumoniae* during ICU stay. *P* value was calculated using independent samples-Mann Whitney U test. In total, data from 100 patients are included in this figure

The dynamics of acquisition of *K. pneumoniae* in the ICUs is shown in Fig. 1. Patients that acquired a carbapenem-susceptible *K. pneumoniae* had their first positive culture approximately four days sooner than patients that acquired a carbapenem-non-susceptible strain of *K. pneumoniae* (*p* < 0.001). However, the acquisition rate of carbapenem-susceptible *K. pneumoniae* was higher with 41.0 per 1000 patient-days at risk (adult ICU: 46.1; ER-ICU: 35.9) compared to the acquisition rate of carbapenem-non-susceptible *K. pneumoniae* that was 24.7 per 1000 patient-days at risk (adult ICU: 22.2; ER-ICU: 27.0).

**Fig. 2:**
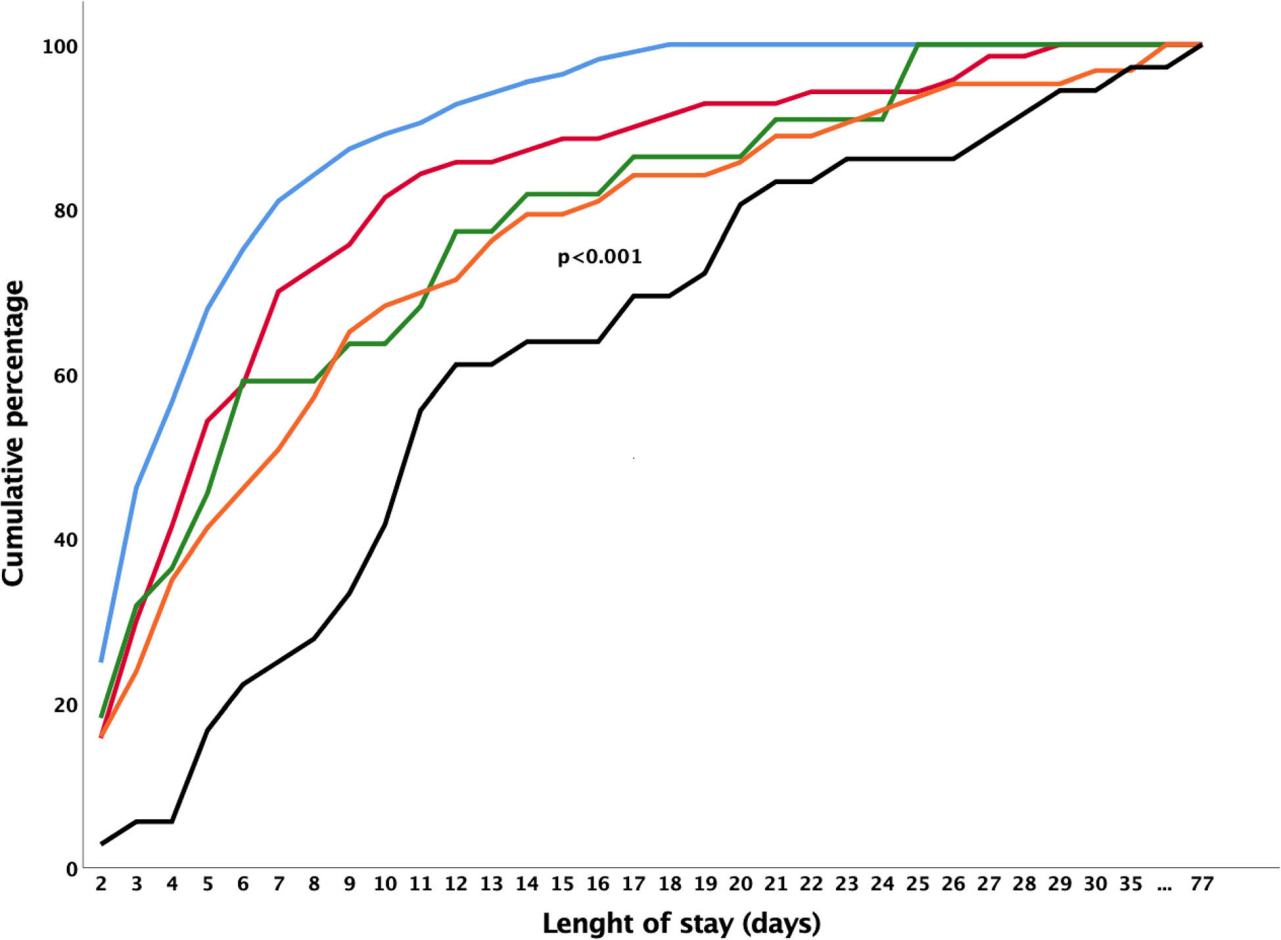
Cumulative percentage of length of stay according to *K. pneumoniae* status. Cumulative length of ICU stay of patients based on their *K. pneumoniae* status. Length of stay (days) represent total days patients were hospitalized in the ICU. The blue line represents patients that were always *K. pneumoniae* negative during their ICU stay. The red line represents patients already positive for carbapenem-susceptible *K. pneumoniae* on the day of admission. The green line represents patients already positive for carbapenem-non-susceptible *K. pneumoniae* on the day of admission. The orange line represents patients that acquired carbapenem-susceptible *K. pneumoniae* during ICU stay and the black line represents patients that acquired carbapenem-non-susceptible *K. pneumoniae* during ICU stay. The length of stay of patients that became positive with carbapenem-non-susceptible *K. pneumoniae* during ICU stay was longer than that of the other groups (Cox regression, *P* < 0.001)

Patient outcomes were clearly associated with *K. pneumoniae* status. Patients who acquired carbapenem-non-susceptible *K. pneumoniae* during ICU stay had a significantly longer length of stay (median [interquartile range (IQR)]: 11 [8–20] days, adjusted Hazard Ratio [aHR]: 2.32 [99% confidence interval (CI): 1.35–3.68], *p* < 0.001, Fig. 2 and Supplementary Table 4) compared to the other groups of patients, of whom ≥80% were discharged from the ICU within 2–13 days. Interestingly, these latter groups included the patients that were always free from *K. pneumoniae*, and patients that already carried *K. pneumoniae* (either carbapenem-susceptible or carbapenem-non-susceptible) at the time of admission to the ICU and patients who became positive for carbapenem-susceptible *K. pneumoniae* during their stay in ICU (Fig. 2).

A longer length of stay was also associated with mechanical ventilation ≥5 days (median [IQR]: 10 [7–15], aHR: 2.79 [CI_99_]: 1.80–4.34], *p* < 0.001, Supplementary Table 4) and use of a urinary catheter ≥5 days (median [IQR]: 8 [5–12], aHR: 3.88 [CI_99_: 2.14–7.04], *p* < 0.001, Supplementary Table 4) during ICU stay.

**Fig. 3:**
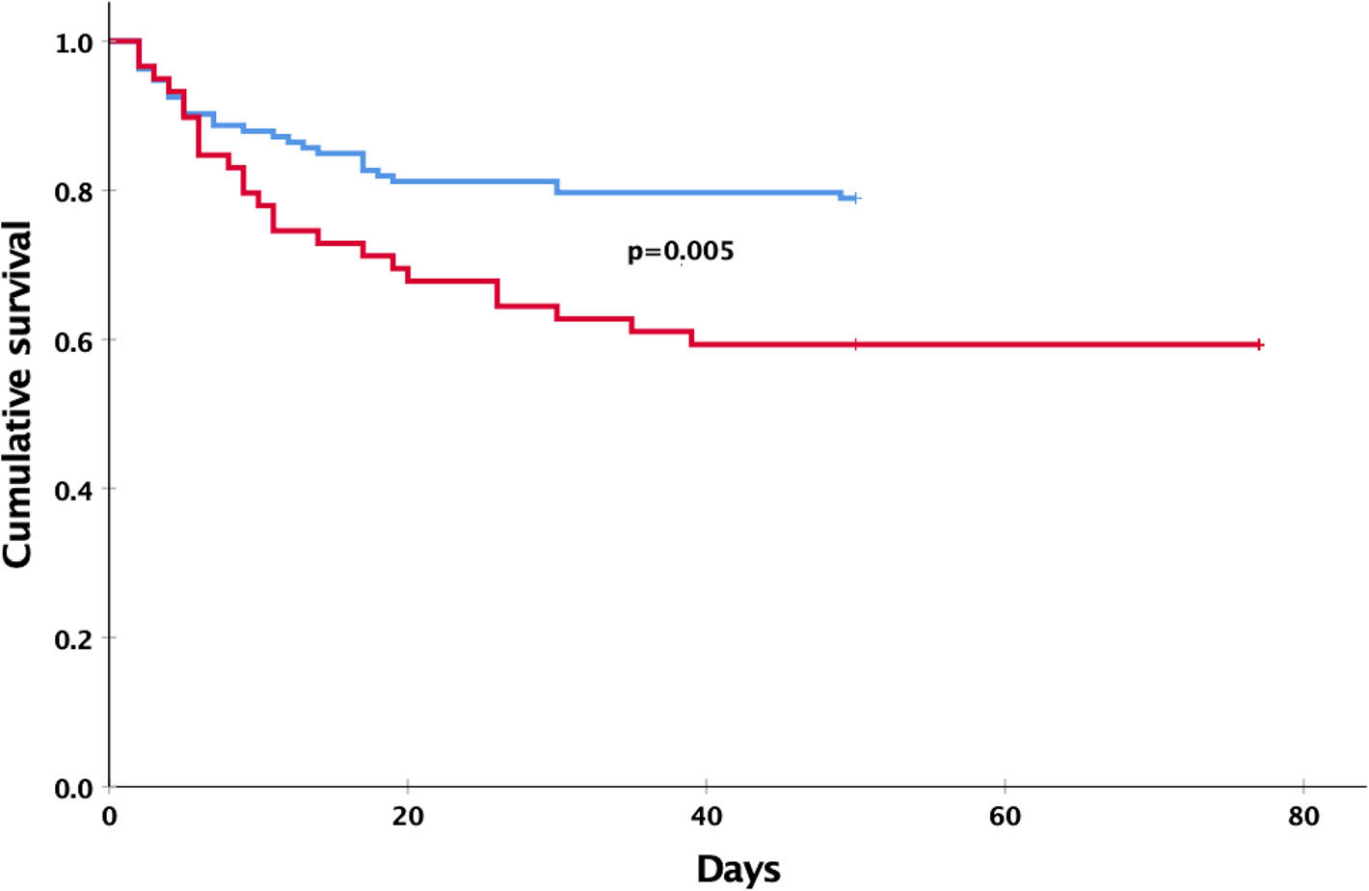
Survival of patients according to their *K. pneumoniae* status. Survival of patients with carbapenem-non-susceptible *K. pneumoniae* (on admission or acquired during ICU stay) (red line) compared with the survival of patients that had carbapenem-susceptible *K. pneumoniae* (on admission or acquired during ICU stay) (blue line) in their screening and/or clinical cultures. *P* value was calculated using logistic regression

However, the acquisition of *K. pneumoniae* was not associated with in-ICU mortality, 30.5% of patients that remained free of *K. pneumoniae* died versus 17.5 and 43.2% of patients that acquired a carbapenem-susceptible or non-susceptible *K. pneumoniae* strain, respectively*,* during their ICU stay (Supplementary Table 5, adjusted Odds Ratio [aOR]: 0.40 [99% CI: 0.14–1.13], *p* = 0.023 and 1.03 [0.36–2.97], *p* = 0.937). Interestingly, the group of patients that carried a carbapenem-susceptible strain of *K. pneumoniae*, either on admission or acquired during ICU stay, had the lowest observed mortality rates (24.3 and 17.5%, respectively), even lower than the 30.5% mortality observed among those patients that were always negative for this species, but this difference did not reach statistical significance. However, when compared to patients that had a carbapenem-non-susceptible isolate of *K. pneumoniae*, either on admission or during their ICU stay, the ICU survival of patients with carbapenem-susceptible strains was significantly higher (aHR: 2.57 [99% CI: 1.07–6.17], *p* = 0.005, Fig. 3). Importantly, the admission SIRS and qSOFA scores of patients with or without *K. pneumoniae* acquisition did not differ (Table 1), indicating that a difference in the risk of dying was not present at the time of ICU admission but emerged later during their ICU stay (SIRS: crude Odds Ratio [cOR]: 1.69 [99% CI: 0.55–5.22], *p* = 0.230; qSOFA: cOR: 1.45 [99% CI: 0.68–3.08], *p* = 0.211, Supplementary Table 5).

**Table 1:** Patient characteristics and outcomes according to their *Klebsiella pneumoniae* status

	Group 1	Group 2	Group 3	Group 4	Group 5	*p* value
	220	70	22	63	37	
Age (years), median (IQR)	46 (33–58)	49 (33–58)	31.5 (25–49)	50 (38–58)	47 (35.5–62)	0.036
Gender (%)						0.533
Male	108 (49.1)	35 (50)	13 (59.1)	35 (55.6)	23 (62.2)	
Female	112 (50.9)	35 (50)	9 (40.9)	28 (44.4)	14 (37.8)	
Underlying diseases (%)						
Cardiovascular						0.556
Yes	10 (4.5)	64 (91.4)	1 (4.5)	5 (7.9)	3 (8.1)	
No	210 (95.5)	6 (8.6)	21 (95.5)	58 (92.1)	34 (91.9)	
Cerebrovascular						0.026
Yes	11 (5.0)	2 (2.9)	3 (13.6)	8 (12.7)	5 (13.5)	
No	209 (95.0)	68 (97.1)	19 (86.4)	55 (87.3)	32 (86.5)	
Chronic kidney disease						0.068
Yes	17 (7.7)	7 (10.0)	0	1 (1.6)	0	
No	203 (92.3)	63 (70.0)	22 (100)	62 (98.4)	37 (100.0)	
Diabetes mellitus						0.138
Yes	12 (5.5)	10 (14.3)	1 (4.5)	6 (9.5)	4 (10.8)	
No	208 (94.5)	60 (85.7)	21 (95.5)	57 (90.5)	33 (89.2)	
Malignancy						0.717
Yes	68 (30.9)	20 (28.6)	4 (18.2)	16 (25.4)	10 (27.0)	
No	152 (69.1)	50 (71.4)	18 (81.8)	47 (74.6)	27 (73.0)	
Indication for ICU admission (%)						0.005
Medical	64 (29.1)	21 (30.0)	10 (45.5)	23 (36.5)	22 (59.5)	
Surgical	156 (70.9)	49 (70.0)	12 (54.5)	40 (63.5)	15 (40.5)	
Referral from (%)						0.378
Other ward this hospital	115 (52.3)	36 (51.4)	15 (68.2)	33 (52.4)	23 (62.2)	
Other hospital	40 (18.2)	14 (20.0)	2 (9.1)	11 (17.5)	10 (27.0)	
Directly from Emergency Unit	65 (29.5)	20 (28.6)	5 (22.7)	19 (30.2)	4 (10.8)	
Antibiotic exposure (pre-ICU admission)						
Any antibiotic (%)	163 (74.1)	54 (77.1)	18 (81.8)	44 (69.8)	32 (86.5)	0.365
Carbapenem (%)	44 (20.0)	3 (4.3)	7 (31.8)	12 (19.0)	13 (35.1)	< 0.001
SIRS Score, (%)						0.598
Score > 2	200 (91.0)	63 (70.0)	22 (100.0)	59 (93.7)	33 (89.2)	
Score < 2	20 (9.0)	7 (10.0)	0	4 (6.3)	4 (10.8)	
qSOFA Score, (%)						0.971
Score ≥ 2	179 (81.4)	56 (80.0)	17 (77.3)	51 (81.0)	31 (83.2)	
Score < 2	41 (18.6)	14 (20.0)	5 (22.7)	12 (19.0)	6 (16.2)	
Procedures (during ICU admission)						
Mechanical ventilation used (%)	199 (90.5)	59 (84.3)	20 (90.9)	57 (90.5)	36 (97.3)	0.314
Mechanical ventilation (days)						< 0.001
≥ 5 days	74 (33.6)	30 (42.9)	12 (54.5)	34 (54.0)	32 (86.5)	
< 5 days	146 (66.4)	40 (57.1)	10 (45.5)	29 (46.0)	5 (13.5)	
Central venous catheter used (%)	193 (87.7)	62 (88.6)	19 (86.4)	53 (84.1)	36 (97.3)	0.343
Central venous catheter (days)						< 0.001
≥ 5 days	96 (43.6)	40 (57.1)	12 (54.5)	42 (66.7)	33 (89.2)	
< 5 days	124 (56.4)	30 (42.9)	10 (45.5)	21 (33.3)	4 (10.8)	
Urinary catheter (%)	220 (100.0)	70 (100.0)	22 (100.0)	63 (100.0)	37 (100.0)	NA
Urinary catheter (days) median (IQR)						
≥ 5 days	112 (50.9)	44 (62.9)	15 (68.2)	46 (73.0)	34 (91.9)	< 0.001
< 5 days	108 (49.1)	26 (37.1)	7 (31.8)	17 (27.0)	3 (8.1)	
Antibiotic therapy (during ICU admission)						
Any antibiotic (%)	217 (98.2)	70 (100.0)	21 (95.5)	62 (98.4)	36 (100.0)	0.474
Carbapenem (%)	110 (49.8)	26 (37.1)	13 (59.1)	29 (46.0)	21 (58.3)	0.179
Outcomes						
Length of stay (days), median (IQR)	4 (2–6)	5 (3–9)	6 (3–13)	7 (4–13)	11 (8–20)	< 0.001
Death (%)	67 (30.3)	17 (24.3)	8 (36.4)	11 (17.5)	16 (44.4)	0.054

Abbreviations: *ICU* Intensive Care Unit; *IQR* Interquartile range; *NS* Non-Susceptible; *qSOFA* quick Sepsis-related Organ Failure Assessment; *SIRS* Systemic Inflammatory Response Syndrome; *S* Susceptible

Group 1: No *K. pneumoniae* on admission and negative for *K. pneumoniae* during ICU admission

Group 2: Carbapenem-S *K. pneumoniae* on admission, no carbapenem-NS *K. pneumoniae* acquisition during ICU admission

Group 3: Carbapenem-NS *K. pneumoniae* on admission, considered as positive during ICU admission (regardless of results of follow-up cultures)

Group 4: No *K. pneumoniae* on admission, acquisition of carbapenem-S *K. pneumoniae* during ICU admission

Group 5: Either no *K. pneumoniae* or carbapenem-S *K. pneumoniae* on admission, acquisition of carbapenem-NS *K. pneumoniae* during ICU admission

Significance was calculated using One-way ANOVA, Pearson Chi Square and Fisher’s Exact Test

A *p*-value less than 0.01 was considered statistically significant

The competing risk estimates analysis also revealed that the incidence of death was higher in patients with a carbapenem-non-susceptible isolate of *K. pneumoniae* (*p* = 0.006), and the incidence of being discharged alive from ICU was higher for patients with a carbapenem-susceptible *K. pneumoniae* (*p* = 0.0005) (Supplementary Figure 2). Patients that acquired a carbapenem-non-susceptible *K. pneumoniae* during ICU stay were more likely to have had prior exposure to antibiotics, especially carbapenems, and they were more likely to have had a medical indication for their admission to the ICU (Table 1).

### Phenotypic and molecular characterization of carbapenem-non-susceptible *K. pneumoniae*

Overall, 99/370 (26.8%) isolates from 59/192 (30.7%) patients were found to be non-susceptible to carbapenems. In addition, one (water from suction connector) out of 31 *K. pneumoniae* isolates cultured from the environment (400 samples taken) was carbapenem-non-susceptible. None of 24 *K. pneumoniae* isolates cultured from HCWs (out of 167 screened) were found to be carbapenem-non-susceptible. Thus, a total of 100 carbapenem-non-susceptible isolates was further subjected to phenotypic and molecular analyses. The phenotypic detection test indicated that 96/100 (96%) isolates produced a MBL. PCRs of carbapenemase genes demonstrated the presence of the *bla*_NDM_ gene in these 96 carbapenem-non-susceptible isolates, including isolates from patients and the one from the environment. None of the 100 isolates was positive for either the *bla*_KPC_ or *bla*_OXA-48_ gene. Four carbapenem-non-susceptible strains apparently contained another resistance mechanism, which was not further investigated, they remained relatively susceptible to carbapenems (MIC meropenem 2–4 mg/L). The string test was positive for only four isolates from three patients, one of whom deceased in ICU.

### Clonal relatedness

**Fig. 4:**
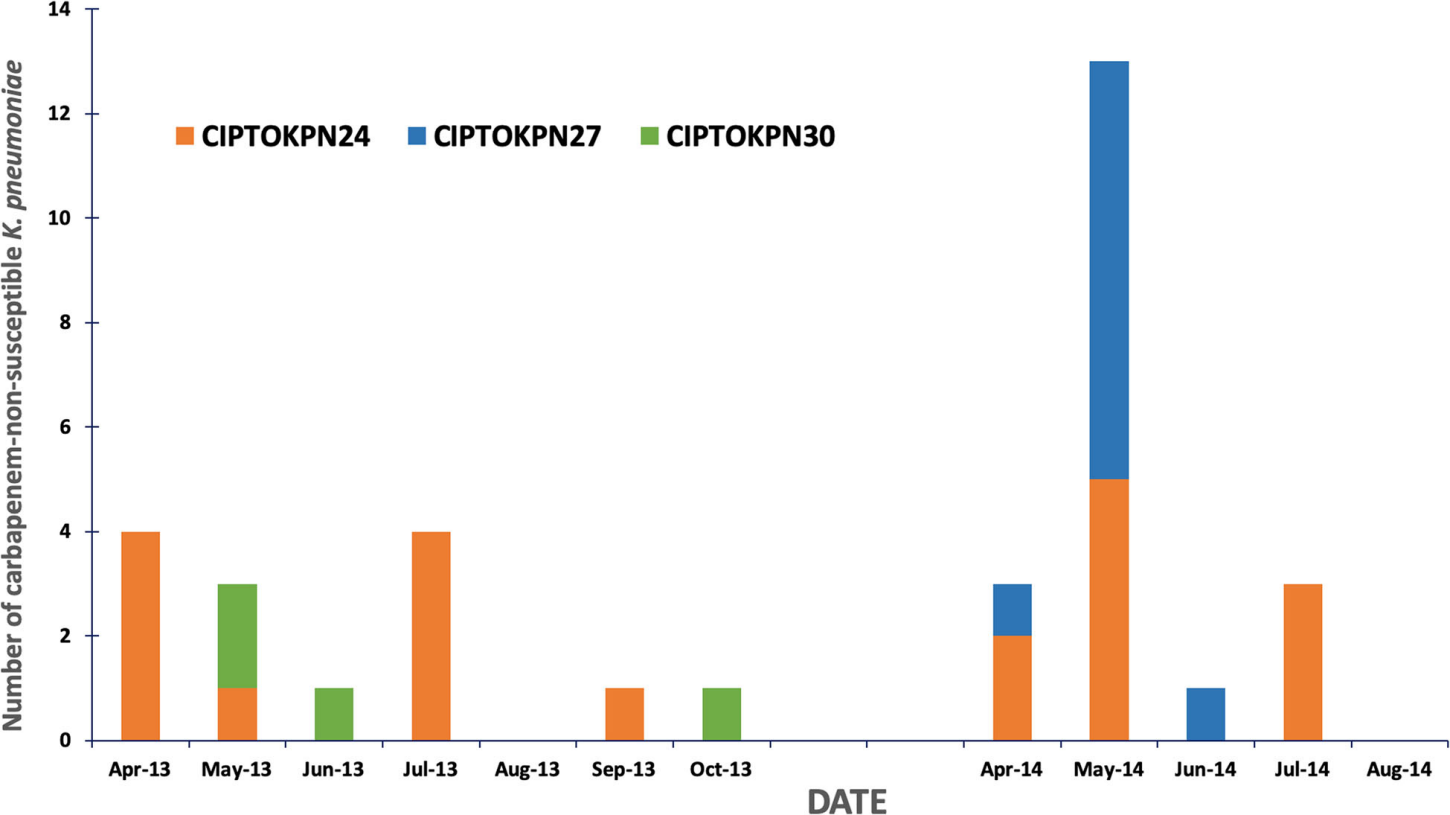
Persistence of prevalent clones of carbapenem-non-susceptible *K. pneumoniae* in ICUs, as determined by Raman spectroscopy typing. Endemicity of the three largest clusters, as determined by Raman spectroscopy, of carbapenem-non-susceptible *K. pneumoniae* in ICUs, April–October 2013 and April–August 2014. The orange bars represent cluster CIPTOKPN24. The blue bars represent CIPTOKPN27. The green bars represent CIPTOKPN30. The x-axis indicates time periods of the study (by week, April 2013-Oktober 2013 and April 2014–August 2014). The y-axis indicates number of isolates

Raman spectroscopy analysis performed for 100 isolates revealed the presence of multiple types within this collection of *K. pneumoniae.* In total, 48 Raman types were identified. There were three major clusters (Supplementary Figure 3), the largest cluster (CIPTOKPN24) consisted of 20 isolates obtained from 13 patients (screening and clinical specimens). Strains belonging to the dominant cluster CIPTOKPN24 were present in both ICUs throughout the study period, whereas other clones seemed to wax and wane with time (Fig. 4).

**Fig. 5:**
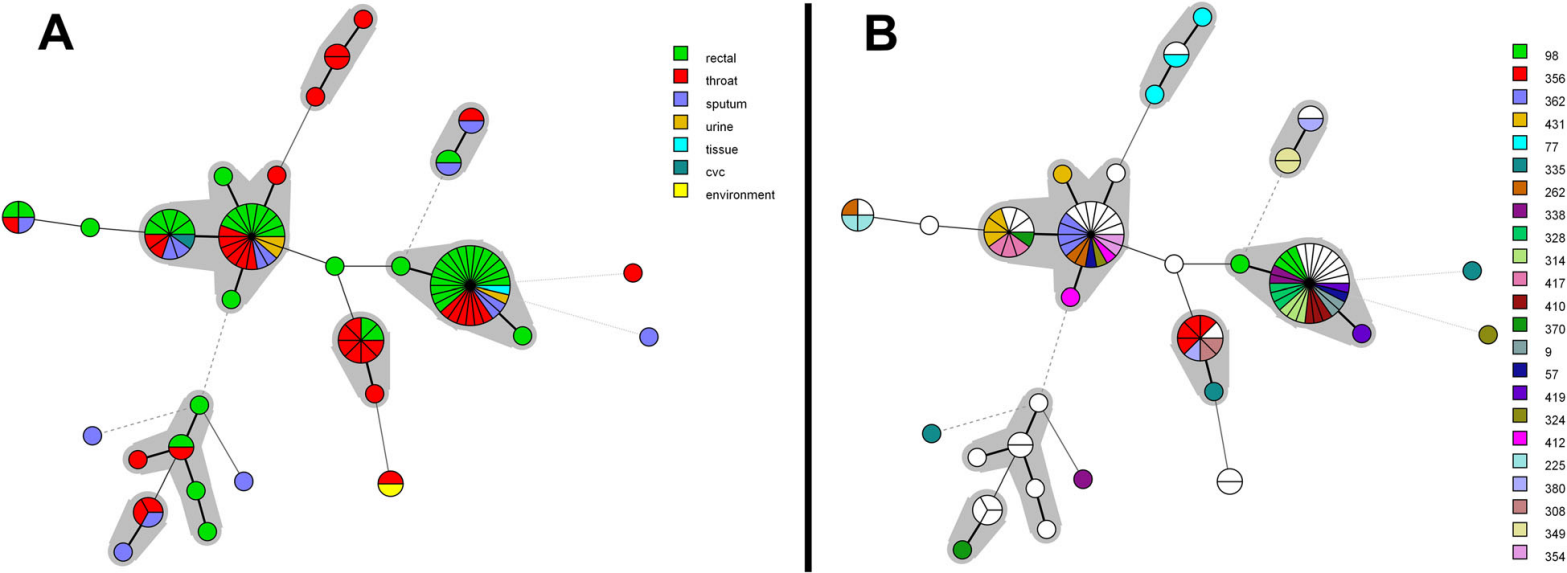
MLVA minimum spanning trees of carbapenem-non-susceptible *Klebsiella pneumoniae.* Minimum spanning tree analysis of *K. pneumoniae* isolates based on clustering at the VNTR loci. Clusters of genotypes differing in only one marker are indicated with a grey background. Panel **a**: Colours correspond to specimens from which *K. pneumoniae* isolates were cultured. Panel **b**: Distribution of genotypes per patient. Each colour, except white, indicates a different patient. Only patients with 2 or more isolates are presented in this manner. Patients that had only one isolate of a carbapenem-non-susceptible *K. pneumoniae* are indicated by the colour white

A total of 97 clinical (two isolates were lost during storage) and 1 environmental isolate were further analyzed using MLVA genotyping, identifying 30 different genotypes (Fig. 5). The most dominant clone accounted for 26.5% (*n* = 26) of all isolates, whereas 19 isolates (19.4%) were of a unique genotype, the remaining 53 isolates belonged to 20 other genotypes. Clustering of strains by Raman spectroscopy into three dominant groups was concordant with clustering by MLVA, e.g. the 20 Raman CIPTOKPN24 strains all belonged to a single MLVA clonal complex. Likewise, the four Raman CIPTOKPN30 strains belonged to a single MLVA clonal complex as did 8/10 CIPTOKPN27 isolates.

## Discussion

This is the first report of a study on the clinical and molecular epidemiology of carbapenemase-producing *K. pneumoniae* in ICUs in Indonesia. These two ICUs can be considered to have endemic carbapenem-non-susceptible *K. pneumoniae* whose acquisition by patients is associated with prolonged ICU stay and, possibly, an increased risk of dying.

The dissemination of *K. pneumoniae* isolates harboring carbapenemase genes, continues unabated, and reports describing these isolates are emerging from different parts of the world, including Southeast Asia [1, 4, 28]. Colonization and infection with carbapenem-resistant *K. pneumoniae* has been reported in Singapore [29]. In Malaysia, the National Surveillance of Antimicrobial resistance found carbapenem resistance rates among *K. pneumoniae* to increase from 0.5% (11,935 isolates tested) in 2010 to 1.6% (27,911 isolates tested) in 2014 [30]. The Philippines Department of Health’s Research Institute reported a rate of 11.9% in 2015 [30]. Morocco [31], Italy [32], and India have likewise shown dramatic increases over time [30]. Similar to these studies, we found that 59/412 (14.3%) of the patients in the ICU carried a carbapenem-non-susceptible *K. pneumoniae*. By screening on ICU admission 5.3% of patients were already colonized with carbapenem-non-susceptible *K. pneumoniae* prior to their admission to the ICU. This suggests that patients may become colonized with such strains elsewhere in the same hospital or in another hospital from which they are referred, or may come with such strain directly from the community, possibly having acquired their strain during a previous healthcare contact or indirectly from exposure to reservoirs or relatives carrying such strains [33].

Screening cultures can, therefore, be considered helpful for early detection and infection control. It may also be useful to guide rational antibiotic use, since previous studies have shown that colonization with a carbapenem-resistant *K. pneumoniae* is a risk factor for subsequent infection [34, 35]. However, during our study, carbapenem-non-susceptible *K. pneumoniae* strains were not isolated from blood cultures (data not shown).

Our data also show that patients may acquire carbapenem-non-susceptible *K. pneumoniae* during ICU stay in the setting of our study and that these acquisitions are associated with significantly longer ICU stay. At the level of significance chosen, the acquisition of *K. pneumoniae* strains, whether carbapenem susceptible or not, was not associated with mortality when compared to patients who remained free of *K. pneumoniae*. In contrast, the study from Dautzenberg et al. (2015) showed patients colonized with carbapenemase-producing Enterobacteriaceae to have on average a 1.79 times higher hazard of dying in ICU than non-colonized patients, primarily because of an increased length of stay [36]. A study in Singapore reported that cases with carbapenem-resistant strains of Enterobacteriaceae had ~ 3.5 times increased odds of fatality adjusted for length of hospital stay [29].

Interestingly, our study shows that the risk of dying among ICU patients who were culture-positive for carbapenem-non-susceptible *K. pneumoniae* on admission or during ICU stay was significantly, 2.57 times, higher than among patients who were culture-positive for a carbapenem-susceptible *K. pneumoniae*. The observed ICU fatality rates were indeed highest (44.4%) among those acquiring carbapenem-non-susceptible *K. pneumoniae* and lowest (17.5%) amongst the patients acquiring carbapenem-susceptible strains of *K. pneumoniae* during their ICU stay. Since risk of mortality during ICU stay is influenced by many factors, as reported by other international findings [32, 37, 38], we cannot readily explain why susceptible *K. pneumoniae* acquisitions seem to be a proxy for protection whereas non-susceptible *K. pneumoniae* acquisitions may be predictive of a fatal outcome. Probably, exposure to carbapenem antibiotics, and the underlying reasons for this, may be important determinants in this respect.

The *bla*_NDM_ gene was the most prevalent carbapenemase gene as it was detected in 96 isolates, including one from the environment. Carbapenem resistance due to *bla*_KPC_-like and *bla*_OXA-48_-like genes was not detected. In South Asia (India and Pakistan) the NDM-1 gene was initially found and currently, this enzyme is by far the most prevalent and widely distributed carbapenem degrading enzyme in the world, including in Southeast Asia [1, 3–6, 8–10, 18, 28–31].

Walsh et al. (2011) have also found the presence of NDM-1 β-lactamase-producing bacteria, including *K. pneumoniae*, from waste seepages samples in Indian community [33]. Taking our study and the recent report on NDM-1 in carbapenem-non-susceptible Enterobacteriaceae from urinay tract samples of hospitalized patients in Surabaya, Indonesia, into account, we suspect that this carbapenemase gene is widespread in hospitals in Indonesia [39].

Carbapenem-susceptible and -non-susceptible *K. pneumoniae* that colonize or infect ICU patients may originate from the patient her/himself, but may also come from contaminated hospital equipment and environment, staff and other patients. In this study *K. pneumoniae* was found in the ICU environment, including one endemic strain that was carbapenem-non-susceptible. Predictably, *K. pneumoniae* was also cultured from throat and rectal swabs of ICU personnel, although none of those isolates was carbapenem-non-susceptible. However, we cannot exclude personnel as a source or vector of *K. pneumoniae* since personnel was only screened once during this study and other body parts (e.g. hands) or clothes were not sampled, limiting the sensitivity of this part of the survey. A recent study in China found that almost 9% of medical personnel in ICU carried multidrug-resistant Gram-negative bacteria on their hands [40]. Transmission of the bacteria may occur with many risk factors involved [1, 3, 41]. Multiple studies reported outbreaks of carbapenem-resistant *K. pneumoniae* that were associated with environmental contamination [42–44]. We performed Raman spectroscopy and MLVA to assess clonal relatedness. These analyses revealed three major clusters by Raman typing, with the largest one (CIPTOKPN24), persisting in both ICUs throughout the whole study period. However, many carbapenem-non-susceptible strains of *K. pneumoniae* cultured in this study were of a unique Raman type or belonged to small clusters that waxed and waned quickly, indicating both endemicity of certain clones in the ICUs but also regular new introductions and rapid loss of many clones over time. This epidemiologic information can and should be applied when designing interventions to reduce the acquisition of carbapenem-resistant *K. pneumoniae* in ICUs in Indonesia and in similar settings elsewhere.

Although not used routinely, Raman spectroscopy typing can be valuable for discriminating types of strains within a species [21–23]. Here we showed typing by Raman spectroscopy to yield *K. pneumoniae* strain clustering compatible with clustering based on MLVA genotyping. However, Raman typing results at a given site cannot be directly compared with results generated or published elsewhere, and are, thus, not easily shared or pooled. Considering the modification that we made to MLVA marker VNTR58, the genotype of the most dominant clone in our study was of genotype “4–2.4–3-4-4.3–1–12-19”. This genotype would translate into “4–3–3-4-5-1-12-19” based on the original MLVA typing method for *K. pneumoniae* [24], but this genotype was not observed by Brink et al. [24]. On the other hand, the second and third most dominant genotype “5–3–3-4-6-1-9-12” and “5–3–3-5-6-1-9-12” match genotype “5–3–3-4-6-1-9-1” and a single locus variant thereof from Brink et al. that involves isolates with MLST sequence type ST147. *K. pneumoniae* ST147 strains belong to a relatively common NDM-positive *K. pneumoniae* lineage and have been found in multiple countries across several continents, almost all of which were isolated from humans [45, 46]. Unlike in the original paper, in our approach the non-integer alleles were considered as separate alleles. As a result, the total number of alleles per marker will increase for several markers and as a result this may benefit the overall discriminatory power of the MLVA method. Indeed, in our study (exceeding the ones reported here), we observed several different genotypes that would have been assigned identical when the non-integer alleles would have been ignored by ‘rounding off’ their values using adjusted and broadened binsets (results not shown).

There were some limitations in this study. First, as no colometric agar plate was used for the screening cultures, overgrowth of carbapenem-susceptible but cefotaxime-resistant isolates could have led to overlooking CNKP. However, all morphologically different colonies were checked, and this was done by trained and experienced microbiology technicians. Colometric media are expensive, therefore, could not be used in this study. Also, cefotaxime-susceptible OXA-48-producing isolates could have been missed with our screening method. Nevertheless, based on the isolates found in the clinical cultures, it is unlikely that these were playing a major role in the epidemiology. Of note, the OXA-48 PCR was not able to detect OXA-54 and OXA-436, but given the results of the phenotypic detection method, and epidemiology in countries nearby, these are also not suspected. Second, only two ICUs in one tertiary care academic hospital participated, which does not permit results to be called representative for all ICUs in Indonesia. Third, the study was performed more than 5 years ago, hence the epidemiology of CNKP in Indonesia may be different now. Finally, the limited rate of sampling of the environment and of personnel (as opposed to patients) may have undervalued their role in the chain of transmission and acquisition of carbapenem-non-susceptible *K. pneumoniae.*

## Conclusions

In summary, this study is the largest to date that describes the characteristics and epidemiology of, and outcome associated with carbapenem-non-susceptible *K. pneumoniae* in ICUs in Indonesia. Colonization or infection with carbapenem-non-susceptible *K. pneumoniae* during hospitalization was independently associated with prolonged LOS in the ICU, and may affect survival during ICU stay. Prevention of colonization by and infection from these multidrug-resistant strains requires interventions directed to source control and limiting the introduction and transmission of such strains to and between patients.

## Supplementary information

**Additional file 1: Table S1.** List of environmental samples. **Table S2.** Amplification primers used for MLVA typing. Modification of the original MLVA typing method of Brink et al.  **Table S3.** Baseline characteristics of 412 patients admitted to the adult or Emergency Room (ER) ICUs, and enrolled in this study. **Table S4.** Variables associated with length of stay among patients with and without carbapenem-non-susceptible *Klebsiella pneumoniae*. **Table S5.** Variables associated with mortality among patients with and without carbapenem-non-susceptible *Klebsiella pneumoniae.*

**Additional file 2: Figure S1.***Klebsiella pneumoniae* carriage of included patients admitted to the ICUs (adult- and ER-ICU) of Dr. Cipto Mangunkusomo General Hospital, Jakarta, Indonesia. **Figure S2.** Plot of the cumulative incidence for ICUs discharge alive and death by carbapenem-susceptible and -non-susceptible *K. pneumoniae*. **Figure S3.** Raman spectroscopy-based cluster analysis of *Klebsiella pneumoniae* isolates from adult- and ER-ICUs.

## Data Availability

The datasets used and/or analysed during the current study are available from the corresponding author on reasonable request.

## Abbreviations

aHR: Adjusted Hazard Ratio
aOR: Adjusted Odds Ratio
ATCC: American Type Culture Collection
CI: Confidence interval
CL: Cloxacillin
CNKP: Carbapenem-non-susceptible *Klebsiella pneumoniae*
cOR: Crude Odds Ratio
CRAN: Comprehensive R Archive Network
DPA: Dipicolinic acid
ER: Emergency Room
EUCAST: European Committee on Antimicrobial Susceptibility Testing
HCW: Healthcare worker
ICU: Intensive care unit
IMP: Imipenemase
IQR: Interquartile range
KPC: *Klebsiella pneumoniae* carbapenemase
LOS: Length of stay
MBL: Metallo-β-lactamase
MIC: Minimum inhibitory concentration
MLVA: Multiple-locus variable number tandem repeat analysis
NDM: New Delhi metallo-β-lactamase
OXA: Oxacillinase
PBA: Phenyl boronic acid
qSOFA: Quick Sequential Organ Failure Assessment
SIRS: Systemic inflammatory response syndrome
TSB: Trypticase soy broth
VIM: Verona integron**-**encoded metallo-β-lactamase
