# Supplementary material for: Clinical impact of endemic NDM-producing Klebsiella pneumoniae in intensive care units of the national referral hospital in Jakarta, Indonesia
Source: Antimicrob Resist Infect Control. 2020 May 11;9:61. doi: 10.1186/s13756-020-00716-7 (PMC7216366; doi:10.1186/s13756-020-00716-7)
Supplement: Supplementary file 1 — Additional file 1: Table S1. List of environmental samples. Table S2. Amplification primers used for MLVA typing. Modification of the original MLVA typing method of Brink et al. Table S3. Baseline characteristics of 412 patients admitted to the adult or Emergency Room (ER) ICUs, and enrolled in this study. Table S4. Variables associated with length of stay among patients with and without carbapenem-non-susceptible Klebsiella pneumoniae. Table S5. Variables associated with mortality among patients with and without carbapenem-non-susceptible Klebsiella pneumoniae. [file 13756_2020_716_MOESM1_ESM.docx]

**Supplementary Table 1. List of environmental samples**

| **Sample site** | **Number of samples** | | | |
| --- | --- | --- | --- | --- |
|  | **Adult ICU** | **ER-ICU** | | |
| Wash basin on ICUs ward | 10 | 5 | | |
| Monitor | 14 | 10 | | |
| Ventilator | 15 | 5 | | |
| Ambu bag | 8 |  | | |
| Stethoscope | 10 | 9 | | |
| Drawer handle bedside cabinet | 21 |  | | |
| Plastic multi-purpose container next to each bed | 18 | 8 | | |
| Stainless steel container | 8 | 15 | | |
| Flowmeter | 15 |  | | |
| Infusion stand | 11 | 8 | | |
| Infusion pump | 9 | 6 | | |
| Bed rails | 20 | 14 | | |
| Tap water (wash basin on ICU ward) | 10 | 8 | | |
| Chart paper on bedside cabinet | 11 | 6 | | |
| Bedside cabinet table | 15 | 11 | | |
| Cleaning room: wash basin | 9 |  | | |
| Cleaning room: sink countertop | 3 |  | | |
| Cleaning room: mug | 2 |  | | |
| Cleaning room: dish rack | 3 |  | | |
| Mattress | 5 | 4 | | |
| Comb | 3 |  | | |
| Water from siphon of wash basin | 10 | 5 | | |
| Water from mug next to each bed | 10 | 6 | | |
| Massage oil | 5 |  | | |
| Chlorine solution after use | 3 |  | |  |
| Cleaning wipes | 3 | 4 |  |  |
| Wall | 3 | 2 |  |  |
| Drawer of bedside cabinet | 3 |  |  |  |
| Water from suction | 7 |  |  |  |
| Suction connector/container | 3 |  |  |  |
| Water from humidifier | 1 |  |  |  |
| Water after cleaning a floor | 2 |  |  |  |
| Floor | 2 |  |  |  |
| Nurse station | 1 | 1 |  |  |

Abbreviations: ER-ICU. Emergency room Intensive Care Unit; ICU, Intensive Care Unit

**Supplementary Table 2. Amplification primers used for MLVA typing.** FAM, VIC, NED and PET represent the different fluorescent labels used for detection of the markers.

| **Marker** | **Forward primer (5’-3’)** | **Reverse primer (5’-3’)** | **Conc (µM)** | **Reference** |
| --- | --- | --- | --- | --- |
| VNTR52 | FAM-TTTGGCGGCAGCGGTTTCCC | GCCAGAAAAAGGCGCGCAGC | 0.4 | (1) |
| VNTR45 | FAM-CGCTGACACATTGACGAAAACAGAGA | ATGAATATTGCCCAGTTTCTGGAACAA | 0.4 | (1) |
| VNTR53 | FAM-CGCAGAAGAAAGCGGAAG | TGTTTTAGGCGCATTCTTACC | 0.4 | (1) |
| VNTR51 | VIC-CCGCCGCGCCATCGTTAGAT | TCAACGCGCCCAGCTGAACC | 0.25 | (1) |
| VNTR60 | VIC-CGGTACGAATCTGTTGGATTAAG | GGCCTTCTTCCGGGTCTAT | 0.2 | (1) |
| VNTR10 | VIC-AGCGCGCAGACGATGAGCAG | AGCCCCGCAGTGGGGTTACT | 0.4 | (1) |
| VNTR27 | NED-CAGCGTCAGCGCCAGACCAA | CCATGGCCGGCCTGTGGTTT | 0.4 | (1) |
| VNTR58 | CGGAAGACGTGGTTGATATG | PET-GCCGAACATATCCTTGATCC |  | This study |

**Modification of the original MLVA typing method of Brink et al. [1]**

In our hands, MLVA marker VNTR58 yielded sizing results that were difficult to interpret as the cumulative result of DNA sequence variations within the amplified product as well as due to size variation of a poly-T stretch in the flanking region of the repeat. To eliminate part of these variations we developed alternative primers (Supplementary Table 2) that amplified a much smaller product with sizing results that were more easily interpreted. Since this approach with new primers invalidated the reference values from Brink et al. for correlating the PCR amplicon sizes to the numbers of repeats in this marker, representative alleles were sequenced by Sanger sequencing to determine the number of repeats [1].

In our collection of isolates, we observed multiple alleles that deviated in size from the expected values as the result of insertions/deletions in the flanking region of the repeat region or in the repeat region itself. Specifically, marker VNTR10 involves a 57 bp repeat region but we also observed amplification products that were 12 bp shorter than the expected sizes. DNA sequence analysis confirmed that this was the result of a 12 bp deletion in the flanking region of the repeat (results not shown). Instead of adjusting the binset for such alleles as described in the original *Klebsiella pneumoniae* MLVA paper [1], we assigned these according to the recommendations for assigning non-integer alleles in human forensics [2]. Consequently, allele n.45 for this marker represents a PCR product size that corresponds to n repeats + 45 additional base pairs. Similarly, in marker VNTR60 (amplifying a 7 bp repeat) n.3 alleles were observed, in marker VNTR45 (amplifying a 12 bp repeat) n.4 alleles were observed and in marker VNTR58 n.1 alleles were observed.

**References**

1.Brink AA, von Wintersdorff CJ, van der Donk CF, Peeters AM, Beisser PS, Stobberingh EE, et al. Development and validation of a single-tube multiple-locus variable number tandem repeat analysis for *Klebsiella pneumoniae*. *PLoS One* (2014) 9(3):e91209. Epub 2014/03/13. doi: 10.1371/journal.pone.0091209. PubMed PMID: 24614534; PubMed Central PMCID: PMCPMC3948817.

2. ISFH TD-CcotECot: DNA recommendations-1994 report concerning further recommendations of the DNA Commission of the ISFH regarding PCR-based polymorphisms in STR (short tandem repeat) systems. *Int J Leg Med* 1994, 107:159-160.

**Supplementary Table 3. Baseline characteristics of 412 patients admitted to the adult or Emergency Room (ER) ICUs, and enrolled in this study.**

|  | Adult ICU | ER-ICU | p value |
| --- | --- | --- | --- |
| Number of patients enrolled | 188 | 224 |  |
| Age (years). median (IQR) | 49 (38-58) | 43 (30-58) | 0.041 |
| Gender |  |  |  |
| Male (%) | 91 (48.4) | 123 (54.9) | 0.188 |
| Female (%) | 97 (51.6) | 101 (45.1) |  |
| Underlying diseases | 126 (54.8) | 104 (45.2) | 0.000** |
| Cardiovascular (%) | 12 (6.4) | 13 (5.8) | 0.806 |
| Cerebrovascular (%) | 10 (5.3) | 19 (8.5) | 0.211 |
| Chronic kidney disease (%) | 9 (4.8) | 16 (17.1) | 0.319 |
| Diabetes mellitus (%) | 15 (8) | 18 (8) | 0.983 |
| Malignancy (%) | 80 (42.6) | 38 (17) | 0.000** |
| Indication for ICU admission |  |  | 0.039 |
| Medical (%) | 54 (28.7) | 86 (38.4) |  |
| Surgical (%) | 134 (71.3) | 138 (61.6) |  |
| Referral from |  |  | 0.000** |
| Other ward this hospital (%) | 144 (76.6) | 78 (34.8) |  |
| Other hospital (%) | 20 (10.6) | 57 (25.4) |  |
| Directly from Emergency Unit (%) | 24 (12.8) | 89 (39.8) |  |
| Antibiotic exposure (before admission to ICU) |  |  |  |
| Any antibiotic (%) | 146 (77.7) | 165 (73.7) | 0.349 |
| Carbapenem (%) | 40 (21.3) | 39 (17.4) | 0.321 |
| SIRS Score. (%) |  |  | 0.992 |
| Score >2 | 172 (91.5) | 205 (91.5) |  |
| Score <2 | 16 (8.5) | 19 (8.5) |  |
| qSOFA Score. (%) |  |  | 0.158 |
| Score >2 | 158 (84.0) | 176 (78.6) |  |
| Score <2 | 30 (16.0) | 48 (21.4) |  |
| Procedures (during ICU admission) |  |  |  |
| Mechanical ventilation (%) | 170 (90.4) | 201 (89.7) | 0.815 |
| Mechanical ventilation (days), median (IQR) | 4 (1.5-9) | 3 (2-7) | 0.591 |
| >5 days (%) | 87 (26.3) | 95 (42.4) | 0.431 |
| <5 days (%) | 101 (53.7) | 129 (57.6) |  |
| Central venous catheter (%) | 166 (88.3) | 197 (87.9) | 0.913 |
| Central venous catheter (days). median (IQR) | 5.5 (3-10) | 5 (3-8.5) | 0.150 |
| >5 days (%) | 106 (56.4) | 117 (52.2) | 0.400 |
| <5 days (%) | 82 (43.6) | 107 (47.8) |  |
| Urinary catheter (%) | 188 (100) | 224 (100) | N/A |
| Urinary catheter (days), median (IQR) | 6 (3-11) | 5 (3-9) | 0.181 |
| >5 days (%) | 118 (62.8) | 133 (59.4) | 0.486 |
| <5 days (%) | 70 (37.2) | 91 (40.6) |  |
| Antibiotic therapy (during ICU admission) |  |  |  |
| Any antibiotic (%) | 188 (100) | 218 (97.3) | 0.034 |
| Carbapenem (%) | 99 (52.7) | 100 (44.6) | 0.105 |
| Outcomes |  |  |  |
| Length of stay (days). median (IQR) | 5 (3-10.75) | 5 (3-8) | 0.024 |
| Death (%) | 52 (27.7) | 67 (29.9) | 0.616 |

Abbreviations: ER-ICU. Emergency Room Intensive Care Unit; ICU, Intensive Care Unit; IQR, Interquartile range; qSOFA, quick Sepsis-related Organ Failure Assessment; SIRS, Systemic Inflammatory Response Syndrome.

**p<0.01

**Supplementary Table 4. Variables associated with length of stay among patients with and without** **carbapenem-non-susceptible *Klebsiella pneumoniae***

|  | **Length of stay** | **Univariate analysis** | | | | | **Multivariate analysis** | | | |
| --- | --- | --- | --- | --- | --- | --- | --- | --- | --- | --- |
|  | **Median (IQR)** | **P** | **cHR** | **99% CI** | | | **p** | **aHR** | **99% CI** | |
|  |  |  |  | **Lower** | **Upper** |  | |  | **Lower** | **Upper** |
| Group (see note below) |  |  |  |  |  |  | |  |  |  |
| Group 1 | 4 (2-6) | <0.001 | 1,00 |  |  |  | | 1.00 |  |  |
| Group 2 | 5 (3-9) | 0.002 | 1.55 | 1.08 | 2.23 | 0.014 | | 1.41 | 0.98 | 2.04 |
| Group 3 | 6 (3-12) | 0.007 | 1.83 | 1.02 | 3.29 | <0.001 | | 3.57 | 1.83 | 6.94 |
| Group 4 | 7 (4-13) | <0.001 | 2.14 | 1.45 | 3.15 | 0.001 | | 1.64 | 1.10 | 2.43 |
| Group 5 | 11 (8-20) | <0.001 | 3.18 | 1.96 | 5.15 | <0.001 | | 2.32 | 1.35 | 3.68 |
| Gender |  |  |  |  |  |  | |  |  |  |
| Male | 5 (3-10) | 0.426 | 0.92 | 0.72 | 1.19 |  | |  |  |  |
| Female | 4 (3-9) |  | 1.00 |  |  |  | |  |  |  |
| Underlying diseases |  |  |  |  |  |  | |  |  |  |
| Cardiovascular |  |  |  |  |  |  | |  |  |  |
| Yes | 8 (5-12) | 0.199 | 1.31 | 0.77 | 2.22 |  | |  |  |  |
| No | 5 (3-9) |  | 1.00 |  |  |  | |  |  |  |
| Cerebrovascular |  |  |  |  |  |  | |  |  |  |
| Yes | 8 (3-14) | 0.026 | 1.54 | 0.93 | 2.55 | 0.851 | | 1.04 | 0.62 | 1.77 |
| No | 5 (3-9) |  | 1.00 |  |  |  | | 1.00 |  |  |
| Chronic kidney diseases |  |  |  |  |  |  | |  |  |  |
| Yes | 4 (3-6) | 0.041 | 0.65 | 0.38 | 1.12 | 0.605 | | 0.89 | 0.50 | 1.58 |
| No | 5 (3-10) |  | 1.00 |  |  |  | | 1.00 |  |  |
| Diabetes mellitus |  |  |  |  |  |  | |  |  |  |
| Yes | 7 (4-14) | 0.108 | 1.34 | 0.84 | 2.14 |  | |  |  |  |
| No | 5 (3-9) |  | 1.00 |  |  |  | |  |  |  |
| Malignancy |  |  |  |  |  |  | |  |  |  |
| Yes | 4 (3-9) | 0.214 | 0.87 | 0.66 | 1.16 |  | |  |  |  |
| No | 5 (3-9) |  | 1.00 |  |  |  | |  |  |  |
| Indication for ICU admission |  |  |  |  |  |  | |  |  |  |
| Medical | 6 (3-12) | <0.001 | 1.52 | 1.15 | 2.01 | 0.893 | | 0.98 | 0.72 | 1.35 |
| Surgical | 4 (3-8) |  | 1.00 |  |  |  | | 1.00 |  |  |
| Referral from |  |  |  |  |  |  | |  |  |  |
| Other ward this hospital | 5 (3-10) | 0.197 | 1.21 | 0.83 | 1.77 |  | |  |  |  |
| Other hospital | 5 (3-9) | 0.151 | 1.18 | 0.88 | 1.59 |  | |  |  |  |
| Directly from Emergency Unit | 5 (3-8) |  | 1.00 |  |  |  | |  |  |  |
| Antibiotic exposure (before admission to ICU) |  |  |  |  |  |  | |  |  |  |
| Any antibiotic | 5 (3-10) | 0.014 | 1.33 | 0.99 | 1.79 | 0.097 | | 1.22 | 0.90 | 1.65 |
| No any antibiotic | 4 (3-7) |  | 1.00 |  |  |  | | 1.00 |  |  |
| Carbapenem | 8 (3-13) | 0.012 | 1.37 | 0.99 | 1.90 | 0.366 | | 1.14 | 0.79 | 1.63 |
| No carbapenem | 5 (3-8) |  | 1.00 |  |  |  | | 1.00 |  |  |
| SIRS score |  |  |  |  |  |  | |  |  |  |
| Score ≥2 | 5 (3-9) | 0.651 | 1.08 | 0.69 | 1.71 |  | |  |  |  |
| Score <2 | 4 (2-7) |  | 1.00 |  |  |  | |  |  |  |
| qSOFA |  |  |  |  |  |  | |  |  |  |
| Score ≥2 | 5 (3-10) | <0.001 | 1.62 | 1.17 | 2.25 | 0.031 | | 1.33 | 0.95 | 1.88 |
| Score <2 | 3 (2-6) |  | 1.00 |  |  |  | | 1.00 |  |  |
| Procedures (during ICU admission) |  |  |  |  |  |  | |  |  |  |
| Mechanical ventilation used | 5 (3-10) | <0.001 | 2.50 | 1.61 | 3.89 | 0.369 | | 1.19 | 0.73 | 1.93 |
| No mechanical ventilation used | 3 (2-4) |  | 1.00 |  |  |  | | 1.00 |  |  |
| Mechanical ventilation (days) |  |  |  |  |  |  | |  |  |  |
| >5 days | 10 (7-15) | <0.001 | 6.33 | 4.59 | 8.73 | <0.001 | | 2.79 | 1.80 | 4.34 |
| <5 days | 3 (2-4) |  | 1.00 |  |  |  | | 1.00 |  |  |
| Central venous catheter used | 5 (3-10) | <0.001 | 2.12 | 1.42 | 3.17 | 0.492 | | 0.88 | 0.53 | 1.45 |
| No central venous catheter used | 3 (2-5) |  | 1.00 |  |  |  | | 1.00 |  |  |
| Central venous catheter (days) |  |  |  |  |  |  | |  |  |  |
| >5 days | 9 (6-13) | <0.001 | 6.30 | 4.62 | 8.59 | 0.015 | | 1.69 | 0.97 | 2.93 |
| <5 days | 3 (2-3) |  | 1.00 |  |  |  | | 1.00 |  |  |
| Urinary catheter used | 5 (3-9) | N/A |  |  |  |  | |  |  |  |
| No urinary catheter used | N/A |  |  |  |  |  | |  |  |  |
| Urinary catheter (days) |  |  |  |  |  |  | |  |  |  |
| >5 days | 8 (5-12) | <0.001 | 9.87 | 6.84 | 14.24 | <0.001 | | 3.88 | 2.14 | 7.04 |
| <5 days | 3 (2-3) |  | 1.00 |  |  |  | | 1.00 |  |  |
| Antibiotic therapy (during ICU admission) |  |  |  |  |  |  | |  |  |  |
| Any antibiotic (%) | 5 (3-9) | 0.005 | 3.20 | 1.10 | 9.33 | 0.302 | | 1.55 | 0.52 | 4.62 |
| No any antibiotic | 2 (2-3) |  | 1.00 |  |  |  | | 1.00 |  |  |
| Carbapenem (%) | 7 (4-12) | <0.001 | 1.78 | 1.37 | 2.32 | 0.566 | | 1.07 | 0.78 | 1.48 |
| No Carbapenem | 4 (2-7) |  |  |  |  |  | | 1.00 |  |  |
| Age, correlation coefficient (r) | 0.081 | 0.154 | 1.00 | 0.99 | 1.00 | 0.655 | | 1.00 | 0.99 | 1.01 |
| Mortality | 6 (3-12) | 0.057 | 1.23 | 0.93 | 1.63 | 0.226 | | 0.87 | 0.65 | 1.17 |

Abbreviation: aHR, adjusted Hazard Ratio; cHR, crude Hazard Ratio; CI, Confidence Interval; ICU, Intensive Care Unit; IQR, Interquartile Range; LOS, Length of Stay; NS, Non-susceptible; S, Susceptible; SIRS, Systemic Inflammatory Response Syndrome; qSOFA, quick Sepsis-related Organ Failure Assessment .

Group 1: No *K. pneumoniae* on admission and negative for *K. pneumoniae* during ICU admission.

Group 2: Carbapenem-S *K. pneumoniae* on admission, no carbapenem-NS *K. pneumoniae* acquisition during ICU admission.

Group 3: Carbapenem-NS *K. pneumoniae* on admission, considered as positive during ICU admission (regardless of results of follow-up cultures).

Group 4: No *K. pneumoniae* on admission, acquisition of carbapenem-S *K. pneumoniae* during ICU admission.

Group 5: Either no *K. pneumoniae* or carbapenem-S *K. pneumoniae* on admission, acquisition of carbapenem-NS *K. pneumoniae* during ICU admission.

A p-value less than 0.01 was considered statistically significant.

**Supplementary Table 5. Variables associated with mortality among patients with and without carbapenem-non-susceptible *Klebsiella pneumoniae***

|  |  | |  | **Univariate analysis** | | | | | | | | | **Multivariate analysis** | | | | | | | | | | | |
| --- | --- | --- | --- | --- | --- | --- | --- | --- | --- | --- | --- | --- | --- | --- | --- | --- | --- | --- | --- | --- | --- | --- | --- | --- |
|  | **Mortality** | | | **p** | | **99% CI** | | | | | **p** | | | | | | **99% CI** | | | | | | |  |
|  | **N** | **%** | | |  | **cOR** | **Lower** | | **Upper** | | |  | | | **aOR** | | | **Lower** | | **Upper** | |  |  |  |
| Group (see Note below) |  |  | | |  |  |  | |  | | |  | | |  | | |  | |  | |  |  |  |
| Group 1 | 67 | 30.5 | | |  | 1.00 | |  | |  | | | |  | |  | | |  | |  | |  |  |
| Group 2 | 17 | 24.3 | | | 0.323 | 0.73 | | 0.33 | | 1.65 | | | | 0.413 | | 0.76 | | | 0.31 | | 1.82 | |  |  |
| Group 3 | 8 | 36.4 | | | 0.569 | 1.31 | | 0.39 | | 4.34 | | | | 0.757 | | 1.17 | | | 0.31 | | 4.43 | |  |  |
| Group 4 | 11 | 17.5 | | | 0.045 | 0.48 | | 0.19 | | 1.23 | | | | 0.023 | | 0.40 | | | 0.14 | | 1.13 | |  |  |
| Group 5 | 16 | 43.2 | | | 0.127 | 1.74 | | 0.68 | | 4.43 | | | | 0.937 | | 1.03 | | | 0.36 | | 2.97 | |  |  |
| Gender |  |  | | |  |  |  | |  | | |  | | |  | | |  | |  | |  |  |  |
| Male | 66 | 55.5 | | | 0.362 | 1.22 | 0.70 | | 2.14 | | |  | | |  | | |  | |  | |  |  |  |
| Female | 53 | 44.5 | | |  | 1.00 |  | |  | | |  | | |  | | |  | |  | |  |  |  |
| Underlying diseases |  |  | | |  |  |  | |  | | |  | | |  | | |  | |  | |  |  |  |
| Cardiovascular |  |  | | |  |  |  | |  | | |  | | |  | | |  | |  | |  |  |  |
| Yes | 9 | 7.6 | | | 0.418 | 1.42 | 0.47 | | 4.31 | | |  | | |  | | |  | |  | |  |  |  |
| No | 110 | 92.4 | | |  | 1.00 |  | |  | | |  | | |  | | |  | |  | |  |  |  |
| Cerebrovascular |  |  | | |  |  |  | |  | | |  | | |  | | |  | |  | |  |  |  |
| Yes | 13 | 10.9 | | | 0.049 | 2.12 | 0.78 | | 5.81 | | | 0.515 | | | 1.35 | | | 0.42 | | 4.35 | |  |  |  |
| No | 106 | 89.1 | | |  | 1.00 |  | |  | | |  | | | 1.00 | | |  | |  | |  |  |  |
| Chronic kidney diseases |  |  | | |  |  |  | |  | | |  | | |  | | |  | |  | |  |  |  |
| Yes | 13 | 10.9 | | | 0.011 | 2.87 | 0.98 | | 8.39 | | | 0.043 | | | 2.60 | | | 0.77 | | 8.78 | |  |  |  |
| No | 106 | 89.1 | | |  | 1.00 |  | |  | | |  | | |  | | |  | |  | |  |  |  |
| Diabetes mellitus |  |  | | |  |  |  | |  | | |  | | |  | | |  | |  | |  |  |  |
| Yes | 11 | 9.2 | | | 0.556 | 1.25 | 0.46 | | 3.40 | | |  | | |  | | |  | |  | |  |  |  |
| No | 108 | 90.8 | | |  | 1.00 |  | |  | | |  | | |  | | |  | |  | |  |  |  |
| Malignancy |  |  | | |  |  |  | |  | | |  | | |  | | |  | |  | |  |  |  |
| Yes | 30 | 25.2 | | | 0.326 | 0.79 | 0.42 | | 1.48 | | |  | | |  | | |  | |  | |  |  |  |
| No | 89 | 74.8 | | |  | 1.00 |  | |  | | |  | | |  | | |  | |  | |  |  |  |
| Indication for ICU admission |  |  | | |  |  |  | |  | | |  | | |  | | |  | |  | |  |  |  |
| Medical | 61 | 51.3 | | | <0.001 | 2.85 | 1.59 | | 5.10 | | | 0.016 | | | 1.81 | | | 0.96 | | 3.40 | |  |  |  |
| Surgical | 58 | 48.7 | | |  | 1.00 |  | |  | | |  | | | 1.00 | | |  | |  | |  |  |  |
| Referral from |  |  | | |  |  |  | |  | | |  | | |  | | |  | |  | |  |  |  |
| Other ward this hospital | 58 | 48.7 | | | 0.798 | 0.94 | 0.48 | | 1.83 | | | 0.187 | | | 0.67 | | | 0.31 | | 1.46 | |  |  |  |
| Other hospital | 30 | 25.2 | | | 0.096 | 1.69 | 0.75 | | 3.80 | | | 0.891 | | | 1.05 | | | 0.41 | | 2.68 | |  |  |  |
| Directly from Emergency Unit | 31 | 26.1 | | |  | 1.00 |  | |  | | |  | | | 1.00 | | |  | |  | |  |  |  |
| Antibiotic exposure (before admission to ICU) |  |  | | |  |  |  | |  | | |  | | |  | | |  | |  | |  |  |  |
| Any antibiotic | 100 | 84.0 | | | 0.011 | 2.05 | 0.99 | | 4.23 | | | 0.320 | | | 1.39 | | | 0.59 | | 3.24 | |  |  |  |
| No any antibiotic | 19 | 16.0 | | |  | 1.00 |  | |  | | |  | | | 1.00 | | |  | |  | |  |  |  |
| Carbapenem | 36 | 30.3 | | | <0.001 | 2.52 | 1.29 | | 4.91 | | | 0.116 | | | 1.65 | | | 0.73 | | 3.76 | |  |  |  |
| No carbapenem | 83 | 69.7 | | |  | 1.00 |  | |  | | |  | | | 1.00 | | |  | |  | |  |  |  |
| SIRS score |  |  | | |  |  |  | |  | | |  | | |  | | |  | |  | |  |  |  |
| Score ≥2 | 112 | 94.1 | | | 0.230 | 1.69 | 0.55 | | 5.22 | | |  | | |  | | |  | |  | |  |  |  |
| Score <2 | 7 | 5.9 | | |  | 1.00 |  | |  | | |  | | |  | | |  | |  | |  |  |  |
| qSOFA |  |  | | |  |  |  | |  | | |  | | |  | | |  | |  | |  |  |  |
| Score ≥2 | 101 | 84.9 | | | 0.211 | 1.45 | 0.68 | | 3.08 | | |  | | |  | | |  | |  | |  |  |  |
| Score <2 | 18 | 15.1 | | |  | 1.00 |  | |  | | |  | | |  | | |  | |  | |  |  |  |
| Procedures (during ICU admission) |  |  | | |  |  |  | |  | | |  | | |  | | |  | |  | |  |  |  |
| Mechanical ventilation used (%) | 119 | 100 | | | N/A |  |  | |  | | |  | | |  | | |  | |  | |  |  |  |
| No mechanical ventilation used (%) | 0 | 0 | | |  |  |  | |  | | |  | | |  | | |  | |  | |  |  |  |
| Mechanical ventilation (days) |  |  | | |  |  |  | |  | | |  | | |  | | |  | |  | |  |  |  |
| >5 days | 74 | 62.2 | | | <0.001 | 2.82 | 1.58 | | 5.02 | | | 0.001 | | | 5.08 | | | 1.46 | | 17.7 | |  |  |  |
| <5 days | 45 | 37.8 | | |  | 1.00 |  | |  | | |  | | | 1.00 | | |  | |  | |  |  |  |
| Central venous catheter used (%) | 116 | 97.5 | | | <0.001 | 7.20 | 1.51 | | 34.34 | | | 0.006 | | | 6.38 | | | 1.13 | | 36.09 | |  |  |  |
| No central venous catheter used (%) | 3 | 2.5 | | |  | 1.00 |  | |  | | |  | | | 1.00 | | |  | |  | |  |  |  |
| Central venous catheter (days) |  |  | | |  |  |  | |  | | |  | | |  | | |  | |  | |  |  |  |
| >5 days | 77 | 64.7 | | | 0.006 | 1.85 | 1.04 | | 3.29 | | | 0.011 | | | 0.28 | | | 0.08 | | 1.02 | |  |  |  |
| <5 days | 42 | 35.3 | | |  | 1.00 |  | |  | | |  | | | 1.00 | | |  | |  | |  |  |  |
| Urinary catheter used (%) | 119 | 100 | | | N/A |  |  | |  | | |  | | |  | | |  | |  | |  |  |  |
| No urinary catheter used (%) | 0 | 0 | | |  |  |  | |  | | |  | | |  | | |  | |  | |  |  |  |
| Urinary catheter (days) |  |  | | |  |  |  | |  | | |  | | |  | | |  | |  | |  |  |  |
| >5 days | 85 | 71.4 | | | 0.005 | 1.91 | 1.05 | | 3.50 | | | 0.601 | | | 1.34 | | | 0.32 | | 5.72 | |  |  |  |
| <5 days | 34 | 28.6 | | |  | 1.00 |  | |  | | |  | | | 1.00 | | |  | |  | |  |  |  |
| Antibiotic therapy (during ICU admission) |  |  | | |  |  |  | |  | | |  | | |  | | |  | |  | |  |  |  |
| Any antibiotic (%) | 119 | 100 | | | N/A |  |  | |  | | |  | | |  | | |  | |  | |  |  |  |
| No any antibiotic | 0 | 0 | | |  |  |  | |  | | |  | | |  | | |  | |  | |  |  |  |
| Carbapenem (%) | 81 | 68.1 | | | <0.001 | 3.16 | 1.75 | | 5.72 | | | 0.005 | | | 2.06 | | | 1.07 | | 3.97 | |  |  |  |
| No Carbapenem | 38 | 31.9 | | |  | 1.00 |  | |  | | |  | | | 1.00 | | |  | |  | |  |  |  |
| Age, median (IQR) (years old) | 50 | (38-60) | | | 0.009 | 1.02 | 1.00 | | 1.04 | | | 0.138 | | | 1.01 | | | 0.99 | | 1.03 | |  |  |  |
| LOS, median (IQR) (days) | 6 | (3-12) | | | 0.058 | 1.03 | 0.99 | | 1.06 | | | 0.367 | | | 0.98 | | | 0.94 | | 1.03 | |  |  |  |

Abbreviation: aOR, adjusted Odds Ratio; cOR, crude Odds Ratio; CI, Confidence Interval; ICU: Intensive Care Unit; IQR, Interquartile range; LOS, Length of Stay; NS, Non-susceptible; S, Susceptible; SIRS, Systemic Inflammatory Response Syndrome; qSOFA, quick Sepsis-related Organ Failure Assessment.

Group 1: No *K. pneumoniae* on admission and negative for *K. pneumoniae* during ICU admission.

Group 2: Carbapenem-S *K. pneumoniae* on admission, no carbapenem-NS *K. pneumoniae* acquisition during ICU admission.

Group 3: Carbapenem-NS *K. pneumoniae* on admission, considered as positive during ICU admission (regardless of results of follow-up cultures).

Group 4: No *K. pneumoniae* on admission, acquisition of carbapenem-S *K. pneumoniae* during ICU admission.

Group 5: Either no *K. pneumoniae* or carbapenem-S *K. pneumoniae* on admission, acquisition of carbapenem-NS *K. pneumoniae* during ICU admission.

A p-value less than 0.01 was considered statistically significant.
