# Supplementary material for: Clinical impact of endemic NDM-producing Klebsiella pneumoniae in intensive care units of the national referral hospital in Jakarta, Indonesia
Source: Antimicrob Resist Infect Control. 2020 May 11;9:61. doi: 10.1186/s13756-020-00716-7 (PMC7216366; doi:10.1186/s13756-020-00716-7)
Supplement: Supplementary file 2 — Additional file 2: Figure S1.Klebsiella pneumoniae carriage of included patients admitted to the ICUs (adult- and ER-ICU) of Dr. Cipto Mangunkusomo General Hospital, Jakarta, Indonesia. Figure S2. Plot of the cumulative incidence for ICUs discharge alive and death by carbapenem-susceptible and -non-susceptible K. pneumoniae. Figure S3. Raman spectroscopy-based cluster analysis of Klebsiella pneumoniae isolates from adult- and ER-ICUs. [file 13756_2020_716_MOESM2_ESM.docx]

**Supplementary Figure 1. *Klebsiella pneumoniae* carriage of included patients admitted to the ICUs (adult- and ER-ICU) of Dr. Cipto Mangunkusomo General Hospital, Jakarta, Indonesia**

**
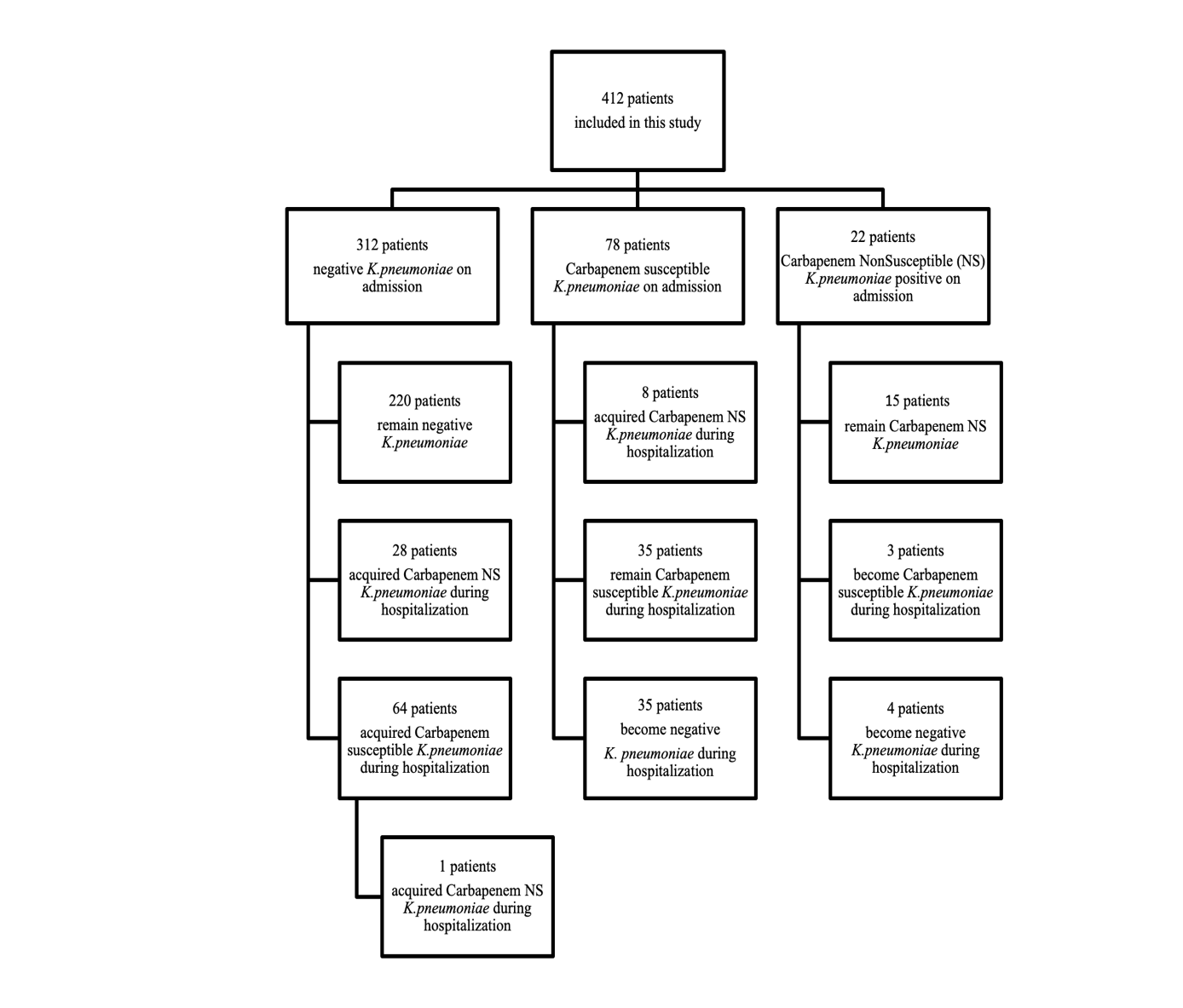
**

**Supplementary Figure 2. Plot of the cumulative incidence for ICUs discharge alive and death by carbapenem-susceptible and -non-susceptible *K. pneumoniae*.**

**
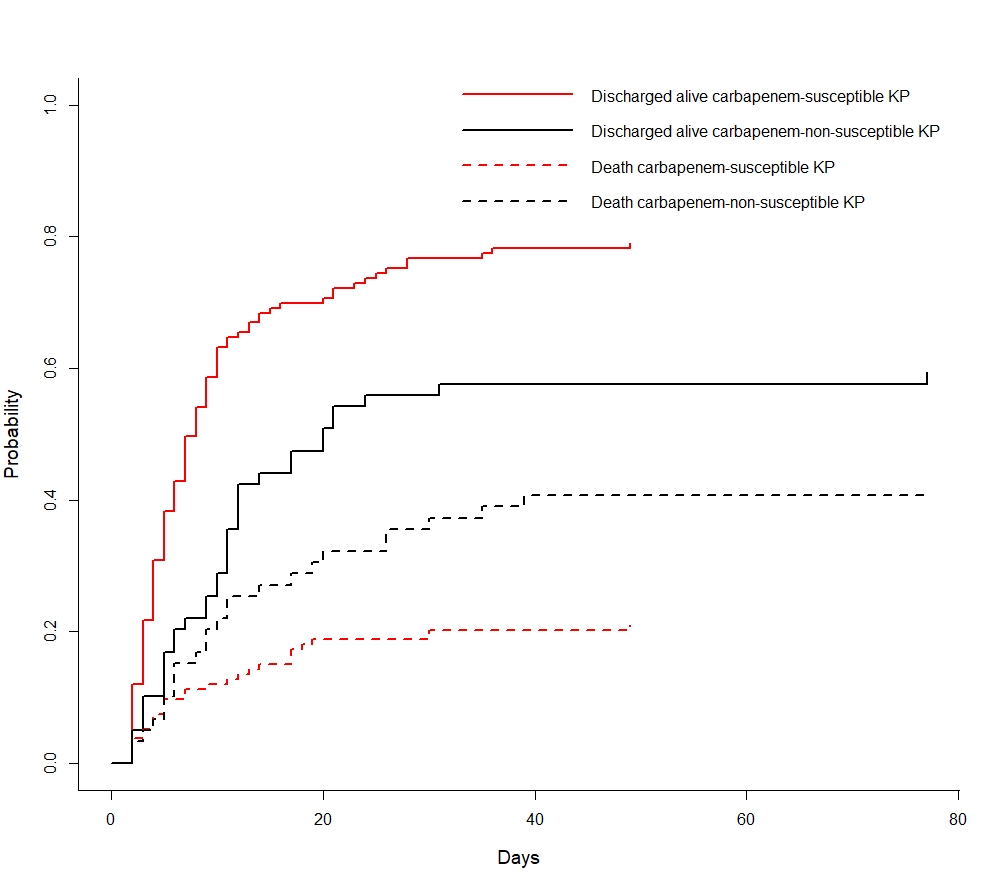
**

**Legend:** Abbreviation: ICU, Intensive Care Unit; KP, *Klebsiella pneumoniae*

The probability of being discharged alive (solid lines) and in-ICUs mortality (dashed lines) among patients harboring carbapenem-susceptible *K. pneumoniae* (black lines) versus patients harboring carbapenem-non-susceptible *K. pneumoniae* (red lines) during their ICU stay. The probability of death in ICUs is significantly higher (p=0.006) among patients with carbapenem-non-susceptible *K. pneumoniae*, conversely the probability of being discharged alive from ICUs is significantly higher for patients with carbapenem- susceptible *K. pneumoniae* (p=0.0005)

**Supplementary Figure 3. Raman spectroscopy-based cluster analysis of *Klebsiella pneumoniae* isolates from adult- and ER-ICUs**


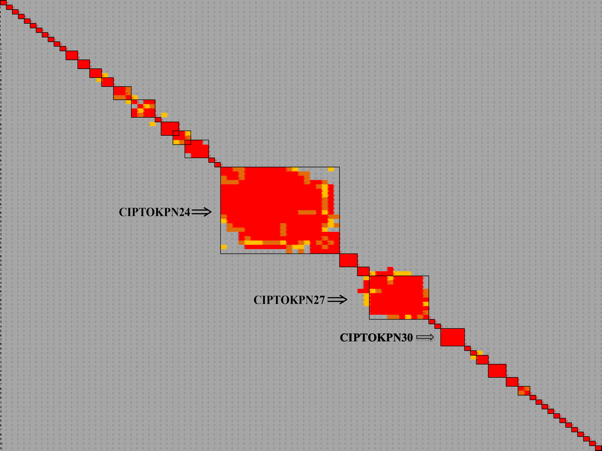


Legend: Raman spectra correlation matrix of carbapenem-non-susceptible *K. pneumoniae* isolates. The similarity between pairs of spectra was calculated using the squared Pearson correlation coefficient (R^2^-values), multiplied by 100 and expressed as a percentage. The similarity threshold for this study was set at 91% (yellow area) so that two isolates with an R^2^ below this threshold were considered to be different and were designated different Raman types. Two isolates with an R^2^-value above 99.5% (red area) were considered indistinguishable and were considered to have the same Raman type. In case of an R^2^-value between of 91% and 99.5% (orange area), these isolates were considered highly related but not identical. There were three dominant cluster CIPTOKPN24, CIPTOKPN27, and CIPTOKPN30.
